# On‐Surface Synthesis of B_3_N_3_‐Substituted Two‐Dimensional Covalent Organic Frameworks with Distinct Pore Sizes and Kagome Band Structures

**DOI:** 10.1002/smll.73718

**Published:** 2026-05-22

**Authors:** Birce Sena Tömekçe, Alireza Nazari Khodadadi, Laura Caputo, Ignacio Piquero‐Zulaica, Martina Corso, Frederik Schiller, J. Enrique Ortega, Jean‐Christophe Charlier, Luigi Vaccaro, Willi Auwärter

**Affiliations:** ^1^ Physics Department E20 TUM School of Natural Sciences Technical University of Munich Garching Germany; ^2^ Laboratory of Green S.O.C. – Dipartimento di Chimica Biologia e Biotecnologie Università degli Studi di Perugia Perugia Italy; ^3^ Institute of Condensed Matter and Nanosciences Université catholique de Louvain (UCLouvain) Louvain‐la‐Neuve Belgium; ^4^ Department of Chemical Sciences University of Naples ‘Federico II’ Napoli Italy; ^5^ Centro de Física de Materiales (CSIC‐UPV/EHU)‐ Materials Physics Center San Sebastian Spain; ^6^ IKERBASQUE Basque Foundation for Science Bilbao Spain; ^7^ Departamento de Física Aplicada Universidad Del País Vasco San Sebastian Spain

**Keywords:** BN‐substitution, covalent organic framework, density functional calculations, on‐surface synthesis, scanning probe microscopy

## Abstract

Covalent organic frameworks (COFs) are a versatile class of materials whose structural, electronic and functional properties can be tailored by selecting the geometry and chemical composition of their linker and spacer moieties. We have synthesized single‐layer B_3_N_3_‐linked 2D COFs with biphenyl and quaterphenyl spacers on Ag(111) and Au(111) under ultra‐high vacuum conditions. The comprehensive experimental characterization combined scanning tunneling microscopy, bond‐resolved atomic force microscopy and photoemission spectroscopy. Density functional calculations revealed differences between the two BN‐substituted COFs and their carbon‐based analogues. The conduction bands of the COFs are primarily derived from electronic states of the spacer units. Introducing B_3_N_3_ linkers into the COFs increases the band gap and reduces frontier band dispersion, effects that can be further modulated by the length of the spacers. Additionally, the site‐selective dehydrogenation of B_3_N_3_ nodes is shown to locally modify the COF's electronic properties. We thus demonstrate the effect of atomically precise B_3_N_3_ substitution on the electronic structure of two distinct kagome systems, through a comparative analysis of isostructural BN and CC substituted COFs. These results establish a new strategy for developing stable, metal‐free COFs with structural diversity and programmable band structures, offering insights into the controlled BN‐doping of low‐dimensional carbon nanostructures.

## Graphical abstract

Single‐layer, 2D BN‐substituted covalent organic frameworks (COFs) with two distinct periodicities are synthesized on noble metal supports. A multi‐method characterization reveals a non‐planar, chiral structure. Band gaps and frontier band dispersion are controlled by linker chemistry and spacer extension. The synthetic strategies presented in this work shed light on the controllable embedding of borazine cores into atomically precise low‐dimensional carbon nanostructures.

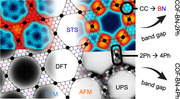

## 1 Introduction

COFs are porous polymeric materials formed by covalent bonding of the molecular building blocks. Thanks to the tunability of their periodic architectures and the versatility of the linkage chemistry, they offer great potential in various applications such as optoelectronics [1, 2, 3, 4], gas separation [5, 6, 7, 8], sensing [9], and energy storage [10]. The incorporation of heteroatoms, the choice of functional groups, and the selected spacer units not only determine the pore size and COF structure but also the degree of π‐conjugation, and hence the electronic properties of COFs [11, 12]. In general, B─N substitution of C─C bonds in π‐conjugated carbon systems is known to modify the electronic structure without structural distortion [13, 14], for example, introducing a band gap to graphene that can be precisely tuned [15, 16, 17, 18]. BN incorporation is further predicted to enhance thermoelectric properties and increase the Seebeck coefficient [19]. Surprisingly, among the many linkages achieved in COFs during the last two decades [20], BN is largely missing. Borazine‐like B_3_N_3_ motifs were reported [21], but exclusively B‐substituted borazine‐based COFs remain elusive.

On‐surface synthesis is a commonly employed bottom‐up strategy to synthesize single‐layer COFs on metallic supports with atomic precision, allowing their fundamental study and characterization under ultra‐high vacuum (UHV) [22, 23, 24, 25, 26, 27, 28, 29, 30, 31, 32], complementing studies at the solid‐liquid interface [33, 34]. The design of the molecular precursors controls the functionalities and structure of the resulting networks. Aiming for BN‐substitution, functionalized borazine precursors promise good control and high versatility [35, 36]. However, to date, only one atomically precise BN‐substituted COF‐like architecture has been achieved, via on‐surface synthesis on Ag(111), featuring hexa‐substituted B_3_N_3_ rings. Yet, its electronic properties remained unexplored [37]. In addition, B_3_N_3_‐substituted amorphous carbon networks [38, 39], B‐ and N‐substituted graphene nanoribbons [40, 41], B‐doped graphene nanoribbons [42, 43], N‐doped covalent porous architectures [29, 30, 44, 45], and a few boroxine incorporated COFs [22, 34] were reported. The latter provide a kagome architecture. These on‐surface synthesis endeavors complement other fabrication strategies to introduce B and N to covalent carbon‐based systems, such as chemical vapor deposition. For example, BN clusters embedded in graphene or extended hybridized hexagonal boron‐nitrogen‐carbon (h‐BNC) sheets have been realized, but they often lack structural control at the atomic level and reproducibility [15, 17, 18, 46, 47, 48].

Overall, despite extensive progress in COF chemistry, functional boron‐doped COFs remain scarce and suffer from limited structural diversity. Control on the band structure to tailor electronic properties has not been demonstrated.

Here, we report the on‐surface synthesis of two single‐layer B_3_N_3_‐substituted 2D COF arrays on Ag(111) and Au(111). The use of biphenyl and quaterphenyl spacer units, respectively, yields distinct pore sizes, B_3_N_3_ areal densities, and electronic structures. The B_3_N_3_ substitution distinguishes these COFs from pure carbon analogues, such as the small pore COF counterpart reported in refs. [23, 24, 25]. A comparison thus allows us to assess the impact of the B_3_N_3_ units on the COF's electronic structure. The use of these B‐substituted B_3_N_3_ nodes provides greater structural property tunability than previously reported hexa‐ and B‐substituted borazine systems [21, 37] and improved physicochemical stability. The COFs are characterized by low‐temperature scanning tunneling microscopy/spectroscopy (LT‐STM/STS), bond‐resolved non‐contact atomic force microscopy (nc‐AFM), and complementary density functional theory (DFT) calculations. STM imaging gives information on the growth characteristics and the network quality. The conduction band was detected as a broad signature in dI/dV spectroscopy for both COFs on both supports. Spatial dI/dV mapping reveals a localization of the conduction band‐derived state on the bi/quaterphenyl spacers, which constitute a kagome lattice. Nc‐AFM shows a dihedral twist between the phenyl rings, with a tilt of 10°–20° with respect to the substrate plane. A band gap of 5.2 eV was determined by combined dI/dV and UV photoemission spectroscopy (UPS) for the BN‐substituted small pore COF on Ag(111), exceeding the value for the respective pure carbon COF by more than 1 eV. DFT yields the band structure, including conduction band, valence band, and electronic gap, for the two pore sizes and different inter‐phenyl twist angles. The computational comparison of the two B_3_N_3_‐substituted COFs and the respective all‐C COFs provides a wealth of information, for example, corroborating the B_3_N_3_‐induced band gap increase and demonstrating a break in symmetry of conduction and valence bands.

## 2 Results and Discussion

**FIGURE 1:**
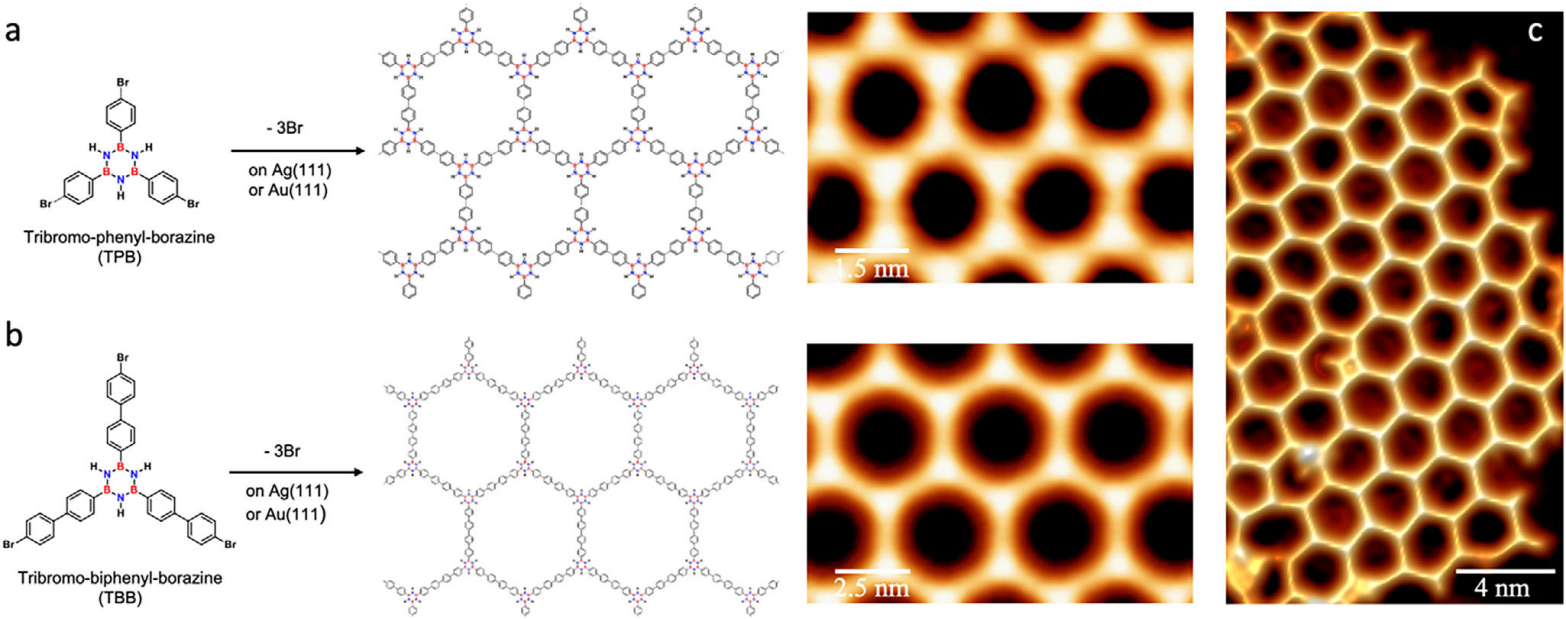
Chemical structures of (a) COF‐BN‐2Ph and (b) COF‐BN‐4Ph synthesized from the precursors tribromo‐phenyl‐borazine (TPB) and tribromo‐biphenyl‐borazine (TBB), respectively, by temperature‐induced Ullmann coupling with STM images of both COFs on Ag(111). (c) STM image of COF‐BN‐2Ph array on Ag(111). (Tunneling parameters for COF‐BN‐2Ph: (a) Current I = 100 pA, bias voltage V_b_ = 0.5 V; (c) Current I = 40 pA, bias voltage V_b_ = 0.02 V, “3D rendering” and Gaussian filter applied; COF‐BN‐4Ph (b): I = 10 pA, V_b_ = 0.2 V).

Both COFs were synthesized by surface‐assisted Ullmann coupling on Ag(111) and Au(111) substrates under UHV conditions. We refer to the COF formed from the tribromo‐phenyl‐borazine (TPB) precursor, having biphenyl bridges, as small pore COF or COF‐BN‐2Ph (following the notation in ref. [49]). The tribromo‐biphenyl‐borazine (TBB) yields the large pore COF, or COF‐BN‐4Ph, featuring para‐quaterphenyl spacer units (see Figure 1). In order to optimize the network quality, we employed different strategies for the growth on the two substrates. On Au(111), a hot deposition approach was used, aiming for direct Ullmann coupling to improve crystallinity [50]. Typical substrate temperatures were 200°C for TPB and 260°C for TBB. To grow COF‐BN‐2Ph on Ag(111), the substrate temperature was kept at around 150°C during deposition to first induce organometallic network formation. Subsequently, a slow heating (∼ 2°C/min) up to 200°C was applied for the conversion to the covalent network [23, 24]. For the fabrication of the large pore COF‐BN‐4Ph on Ag(111), the substrate was held at 250°C during deposition, followed by slow heating to 300°C to induce covalent coupling. An organometallic network formation was observed at 260°C (see Figure S1). The preparations of both COFs on Ag(111) were followed by post‐annealing at around 420°C for 10 min to desorb the cleaved off Br atoms from the surface.

Following these procedures, we successfully prepared COF‐BN‐2Ph and COF‐BN‐4Ph arrays on Ag(111) (Figure 1) and Au(111) (Figure S2). The periodicities of the COF‐BN‐2Ph and COF‐BN‐4Ph determined from STM data are 2.32 ± 0.09 nm and 3.78 ± 0.15 nm, respectively, in reasonable agreement with the calculated unit cell dimensions (see Figure S3, 2.31 nm and 3.82 nm). The COF‐BN‐2Ph periodicity is similar to literature reports for COF‐CC‐2Ph (2.3 nm, ref. [23]; 2.41 nm, ref. [24]). The distances between the borazine‐like B_3_N_3_ nodes amount to 1.3 ± 0.07 nm for COF‐BN‐2Ph and 2.2 ± 0.07 nm for COF‐BN‐4Ph. For COF‐BN‐2Ph, the value matches the distance between phenyl nodes of the isostructural COF‐CC‐2Ph [23]. The two networks yield areal B_3_N_3_ densities of 0.43 and 0.16 / nm^2^, respectively. While the B_3_N_3_ nodes constitute a hexagonal lattice, the bi‐ and quaterphenyl spacer units are arranged in a kagome lattice (Figure S3). To our knowledge, the pore size of COF‐BN‐4Ph exceeds all values known from the literature for COFs synthesized on solid supports.

For both COF types, a higher structural order was achieved on Ag(111) than on Au(111), as reflected in a statistical analysis of the pore shapes (hexagons versus other polygons) provided in Figure S4. Such a reduced structural quality of covalent architectures on Au(111) compared to Ag(111) was previously attributed to the lack of organometallic network formation [25] and faster precursor diffusion [51], promoting the formation of defects and a wider distribution of pore shapes. Therefore, from here on, we will focus on data obtained on Ag(111). Figure S5 shows examples of extended crystalline domains of COF‐BN‐2Ph and COF‐BN‐4Ph. The former includes a 5 × 7 array of hexagonal pores, the latter a 3 × 4 array with hexagonal order. COF‐BN‐2Ph forms more compact domains, whereas COF‐BN‐4Ph arrays are more branched and disordered. Both COFs grow in multiple rotational domains with a wide‐angle distribution (see Figure S6). The array sizes exceed the ones reported for related systems, such as COF‐BO‐2Ph [22]. It was demonstrated that even arrays limited to a few pores can provide valuable insights, representing electronic features of extended COFs [22].

**FIGURE 2:**
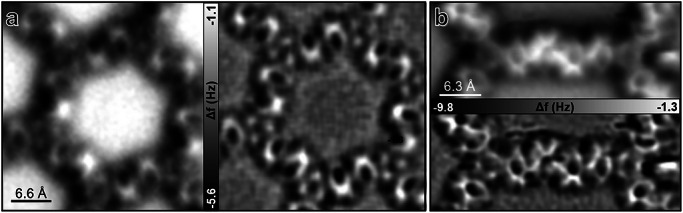
Nc‐AFM, Δf images of (a) COF‐BN‐2Ph and (b) COF‐BN‐4Ph. Corresponding Laplacian filtered, Gaussian smoothened, and contrast‐inverted images are shown in the right panel in (a) and in the bottom panel in (b), respectively.

Nc‐AFM measurements with CO‐functionalized tips were performed to reveal the chemical structure of the COFs by allowing bond resolution [52]. Figure 2 shows nc‐AFM frequency shift (Δf) images of a complete hexagon unit of the small pore COF (Figure 2a) and a hexagon edge of the large pore COF (Figure 2b). The nc‐AFM data clearly confirm the covalent bond formation, yielding biphenyl (for COF‐BN‐2Ph) and quaterphenyl spacer units (for COF‐BN‐4Ph), respectively, e.g., excluding an organometallic character of the networks. Furthermore, the spacer units are not “planar”, i.e., they feature phenyl rings tilted out of the surface plane. Specifically, neighboring phenyl rings show an opposite tilt. For COF‐BN‐4Ph, the two middle phenyls exhibit a larger tilt angle compared to the phenyls adjacent to the B_3_N_3_ linkers. Based on the experimental nc‐AFM contrast and a comparison to simulated AFM images, we estimate that the out‐of‐plane tilt angle of the middle phenyls relative to the substrate plane is about 20° (see Figure S7). Such an angle of roughly 20° was proposed for similar structures, including poly‐para‐phenylene chains on Ag(111) [53, 54]. Moreover, a closer look at the typical hexagonal pore structure of both COFs reveals a chiral character, induced by a concerted, unidirectional tilt of the phenyl rings adjacent to the B_3_N_3_ nodes. This chirality is translated from single nodes to pores and to the network domains (Figure S8). Unlike the phenyl rings, the B_3_N_3_ linkers show a 3‐fold symmetry at this tip‐sample distance with N─H sites appearing bright (see Figure 2) and are therefore aligned parallel to the substrate plane (*vide infra*).

**FIGURE 3:**
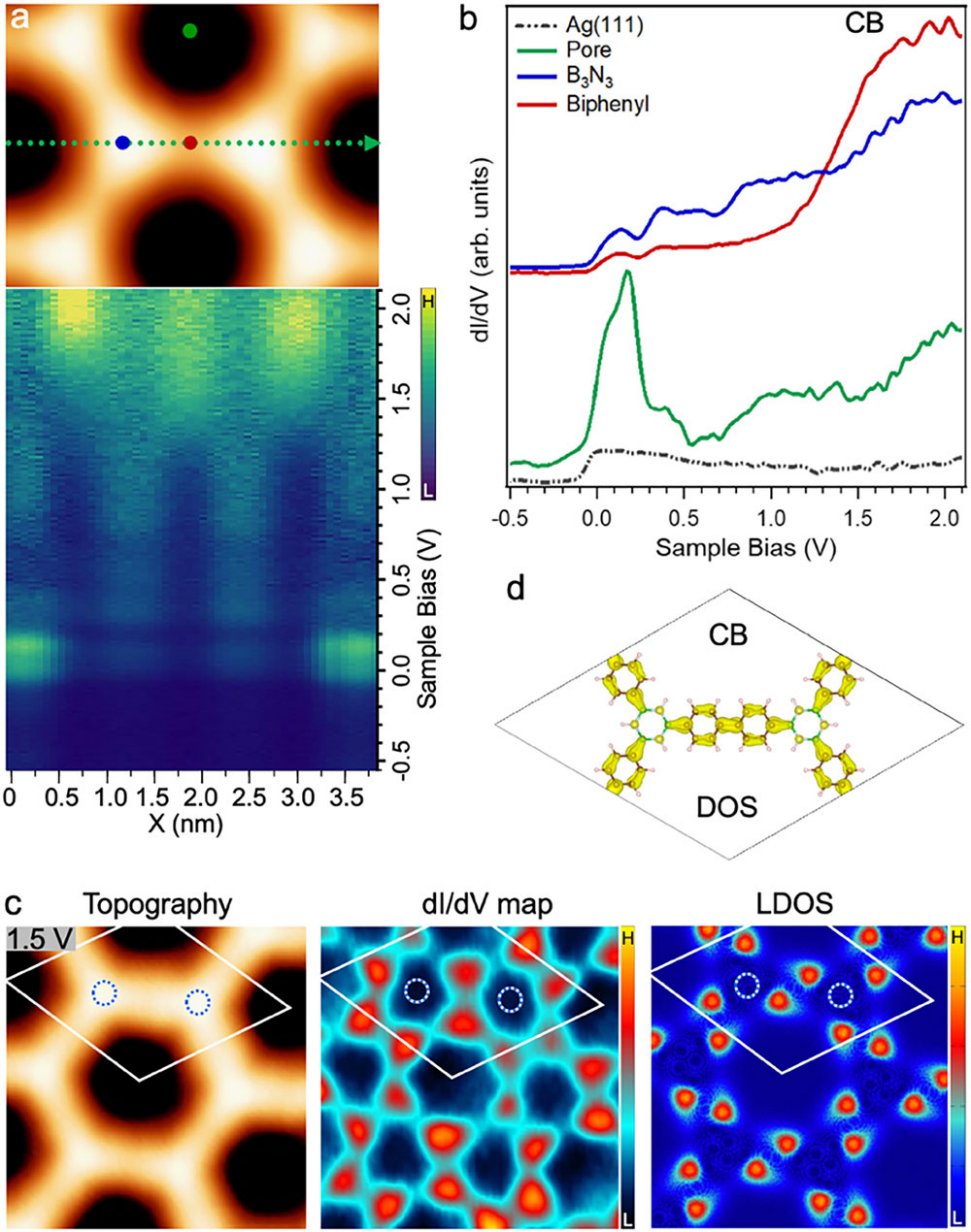
Electronic structure of COF‐BN‐2Ph on Ag(111). (a) STS map representing spectra recorded along the dashed green line in the STM image on top. (Image size: 3.9 nm x 3.0 nm, I = 10 pA, V_b_ = 0.2 V, tip stabilization parameters: 90 pA, 2.1 V, modulation amplitude: 40 mV). (b) Individual spectra selected from the STS map, representing distinct positions (marked by colored dots in (a)). A spectrum acquired on the clean Ag(111) surface is provided as a reference. (c) STM images (left panel), corresponding, simultaneously recorded constant current dI/dV map (middle panel), and calculated LDOS map (right panel, energy integration from 1.58 to 1.88 eV, compare Figure 6a. See Methods section for details). The white rhombi outline the COF unit cell (pore centers at corners), and the blue dashed circles highlight positions of the B_3_N_3_ nodes. (Image scale: unit cell side length 2.32 nm, I = 180 pA. Linear color scales: H and L mark high and low intensity (high and low electron density), respectively. The electron density varies from 0 to 2.5e^−06^ electrons/Bohr^3^. (d) DOS plot for free‐standing COF‐BN‐2Ph unit cell representing the CB (see text for discussion). The isosurface represents a value of 0.05 states/Bohr^3^*Ry.

**FIGURE 4:**
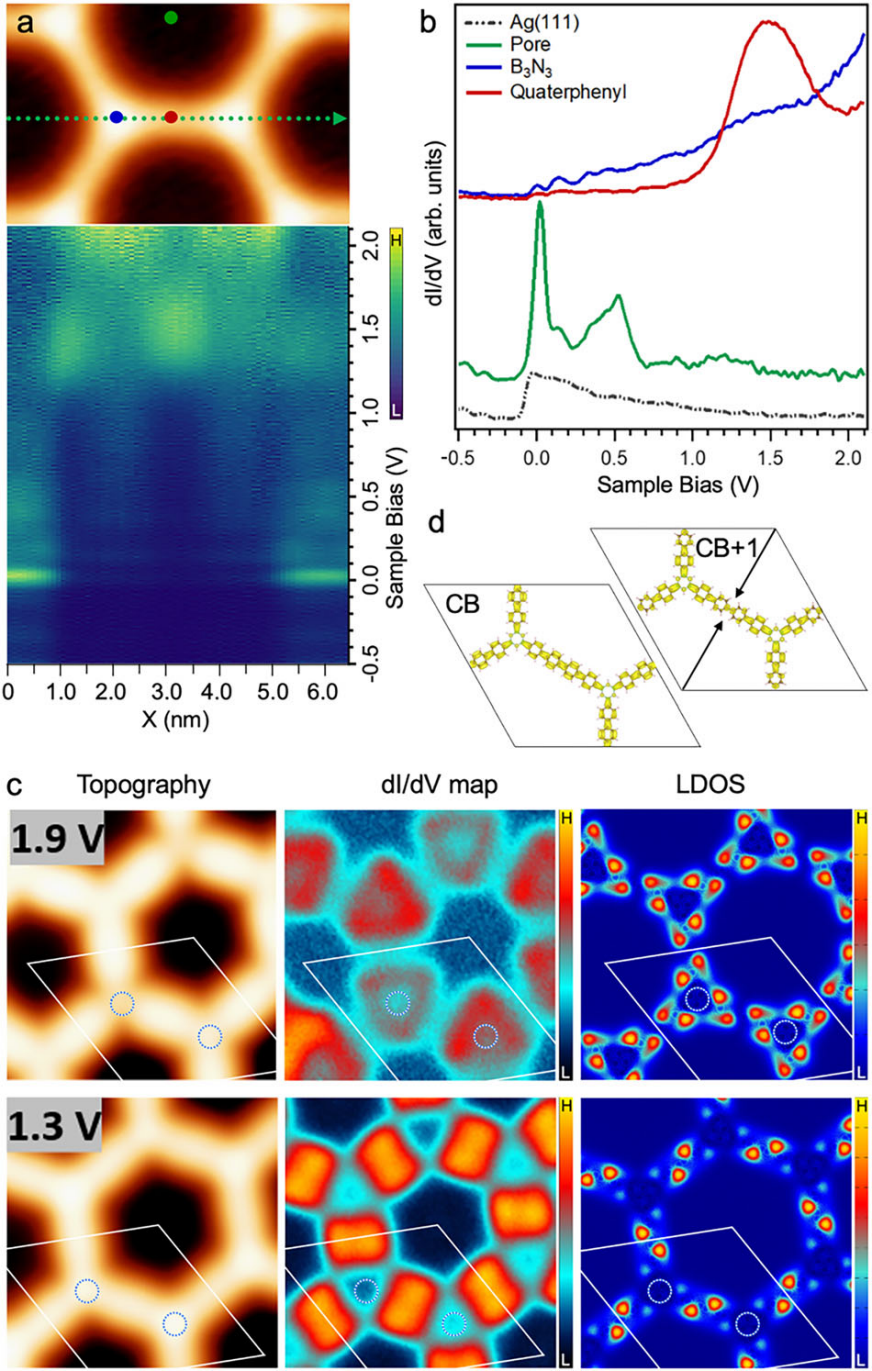
Electronic structure of COF‐BN‐4Ph on Ag(111). (a) STS map representing spectra recorded along the dashed green line in the STM image on top. (Image size: 6.4 × 4.1 nm, I = 20 pA, V_b_ = 0.1 V, tip stabilization parameters: 90 pA, 2.1 V, modulation amplitude: 30 mV). (b) Individual spectra representing distinct positions (marked by colored dots in (a)) (Tip stabilization parameters: 90 pA, 2.1 V, modulation amplitude: 30 mV). (c) Voltage‐dependent STM images (left column), corresponding, simultaneously recorded constant current dI/dV maps (middle column), and calculated LDOS (right column; CB, bottom panel: energy integration from 1.36 to 1.61, CB+1, upper panel: energy integration from 1.81 to 2.21 eV, compare Figure 6c. See Methods section for details). The rhombi (in white) represent the COF unit cell (pore centers at corners) and the blue dashed circles highlight positions of the B_3_N_3_ nodes. (Image size: 6.0 × 6.0 nm, I: 100 pA. Linear color scales: H and L mark high and low intensity (high and low electron density), respectively. The electron density varies from 0 to 1.8e^−06^ electrons/Bohr^3^ (CB) and from 0 to 1.2e^−06^ electrons/Bohr^3^ (CB+1). (d) DOS plot for free‐standing COF‐BN‐4Ph unit cell representing the first CB (left panel) and the second CB (right panel, CB+1). The arrows mark a nodal plane with reduced DOS near the center of the quaterphenyl (see text for discussion). The isosurfaces represent a value of 0.05 states/Bohr^3^*Ry. An enlarged view is provided in Figure S16 for improved clarity.

Next, the electronic properties of the COF arrays were characterized by STS, including dI/dV mapping, and complementary DFT modeling. We first focus on COF‐BN‐2Ph and COF‐BN‐4Ph on Ag(111) (Figures 3 and 4, respectively) and subsequently discuss the electronic structure of both COFs on Au(111) (Figure S9). The STS map in Figure 3a represents spectra recorded along a line from pore center to pore center, crossing a first B_3_N_3_ node (marked by a blue circle in the top panel showing the respective STM image), the biphenyl spacer (marked by a red circle), and a second B_3_N_3_ node. Centered on the biphenyl spacer, a broad spectral feature is detected at bias voltages from 1.3 to 2.1 V and attributed to the conduction band (CB) states (*vide infra*). The respective single spectrum (colored red) is displayed in Figure 3b. Even though the peak is not included completely in the accessible bias voltage range, additional measurements (see Figure S9) attribute the typical peak position to about 1.8 V. Here, we do not apply bias voltages exceeding ≈2.1 V to avoid dehydrogenation of the N sites of the COF (*vide infra*). In the STS map, the maxima adjacent to the central one, at a similar bias voltage, originate from the CB contribution of the adjacent biphenyl spacers (compare Figure S10). At the B_3_N_3_ node, no pronounced, peak‐like signature is observed in the STS map and the corresponding individual spectrum (blue, Figure 3b), but a smooth contribution extends from 2.1 V down to the Fermi level (0 V). For the occupied states regime (negative bias voltages), no spectroscopic features are detected on either the B_3_N_3_ nodes or on the biphenyl spacers by STS down to −2.8 V (see Figure S11). The spectra on the pores reveal the characteristic signature of a confined Ag(111) surface state (see Supporting Information for discussion).

To provide more information on the spatial distribution of the CB state, a dI/dV map representing the CB feature is shown in Figure 3c, together with the corresponding STM image. The CB state is localized transverse to the biphenyl bridges in a dumbbell form, whereas the B_3_N_3_ nodes do not contribute, i.e., appear “dark” (see Figure 3c). The spatial extension of the CB state explains the emergence of the “outer two” CB lobes appearing in the line STS map toward the pore (see Figure 3a). A series of dI/dV maps covering the voltage range of the CB feature is shown in Figure S12, demonstrating that the findings described above apply equally for energies throughout the CB. Figure 3d shows an isosurface plot of the electronic density of states of the CB band of free‐standing COF‐BN‐2Ph, reflecting a non‐planar biphenyl arrangement as observed in the experiment. First, the calculations confirm a prominent electronic contribution of the CB on the biphenyl bridge. Second, this spatial density of states (DOS) distribution does not vary for different energies across the CB band (not shown). And third, a pronounced reduction of the DOS at the B_3_N_3_ node is apparent, in stark contrast to the all‐C COF‐CC‐2Ph (see Figure S13). These properties are intrinsic to the COF's design, even though the presence of the metallic substrate influences the absolute energy levels, for example, through screening and work function alignment (*vide infra*).

With Figure 4, we proceed to the analogous discussion of the large network, COF‐BN‐4Ph, again on Ag(111). The STS map shown in Figure 4a reveals a broad spectral feature centered on the quaterphenyl spacer (red marker in STM image), representing the CB. The CB peak is centered at a voltage of about 1.5 V, as seen from the individual spectrum (red) in Figure 4b. On the B_3_N_3_ nodes, a smooth spectral contribution extends from 2.1 V down to the Fermi level, reminiscent of the situation for the COF‐BN‐2Ph. Again, no spectroscopic features are discernible in the occupied states regime of the network, i.e., a potential valence band contribution is not detected in the probed bias voltage range (down to −1.2 V, not shown). Interestingly, an additional unoccupied state is observed at voltages exceeding the CB, at ∼2.1 V (see Figure 4a). This second state has a node at the center of the quaterphenyl spacers, where the first CB state features an antinode. A similar characteristic was reported for kinked poly‐paraphenylene oligomers [54, 55] and is reminiscent of the first two stationary states of a particle in a box, implying electron confinement in the quaterphenyl segments of COF‐BN‐4Ph, induced by the linkers. In the pores, a step‐like increase in the density of states is observed, with a superimposed peak structure, reflecting the confinement of the Ag(111) surface state. Details on the confined electronic states are provided in Figure S14. Two selected dI/dV maps, including a COF‐BN‐4Ph hexagon, are shown in Figure 4c (see Figure S15 for additional maps at different voltages). At 1.3 V, a pronounced contrast is observed. This CB state is localized on the quaterphenyls, consistent with the STS data, and the B_3_N_3_ nodes show a reduced intensity. At an intermediate voltage of 1.7 V, in between CB and the second conduction band (CB+1), we do not observe a considerable contrast variation on the COF (see Figure S15). On the contrary, a pronounced contrast emerges at 1.9 V, which is “inverted” in comparison to the maps at lower voltages. Specifically, around the B_3_N_3_ linker, an increased signal intensity with triangular symmetry is observed, and the intensity above the centers of the quaterphenyl bridges is reduced. Both observations are in line with the point spectra (see Figure 4a,b). Figure 4d shows the calculated density of states for the CB (left panel) and CB+1 (right panel). As for COF‐BN‐2Ph, key features of the experiments are reflected in the calculations. Importantly, the qualitative match of the spatial DOS distribution with the dI/dV contrast corroborates the assignment of the first unoccupied spectral feature to the first CB and the second unoccupied spectral feature to the second CB (i.e., CB+1, *vide infra*).

**FIGURE 5:**
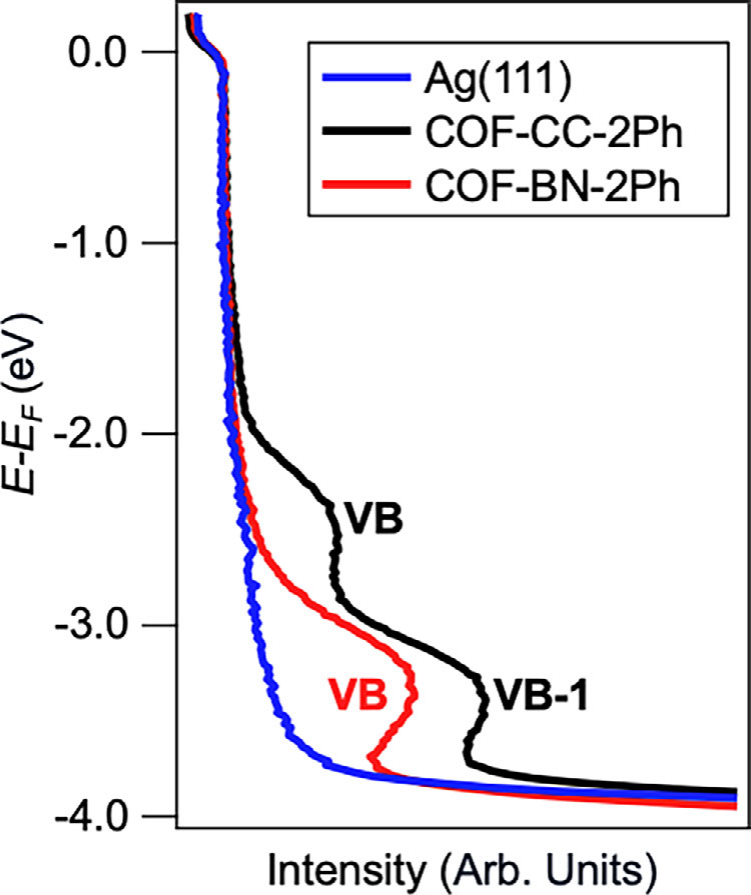
UPS EDCs comparing COF‐BN‐2Ph and COF‐CC‐2Ph with a reference of bare Ag(111). The EDCs are extracted at k_y_ = 1.45 Å^−1^ along the $\bar{\Gamma K}\;$ direction of Ag(111) (see Figure S17 and Methods section for more details). The full angle‐resolved photoemission intensity maps are provided in Figure S17.

Figure S9 provides additional STS data of COF‐BN‐2Ph and COF‐BN‐4Ph on Ag(111) and Au(111). The CB feature centered on the spacer units is clearly detected for all four systems. On Au(111), the respective peaks are upshifted by about 0.8 V compared to Ag(111). This shift is assigned to the work function difference of the two supports [Au(111): 5.26 eV [56]; Ag(111): 4.46 eV [57]]. Furthermore, comparing small and large COFs on the same support, the CB minimum of COF‐BN‐2Ph is about 0.3 V higher than for COF‐BN‐4Ph, and the CB width of COF‐BN‐2Ph is larger compared to that of COF‐BN‐4Ph. The last effect is nicely reflected in the voltage‐dependent dI/dV maps: While for COF‐BN‐2Ph the nodal structure does not change in the bias voltage range of 0.6 V (Figure S12), two distinct nodal structures are observed for COF‐BN‐4Ph in the very same bias voltage range of 0.6 V (Figure 4c, Figure S15). Interestingly, the electronic properties of COF‐BN‐2Ph/Ag(111) can be compared to the published STS results on the all‐C COF‐CC‐2Ph/Ag(111), where the CB was detected at 1.62 V [24]. Accordingly, this comparison reveals a CB band upshift of~0.2 V upon replacement of the benzene‐based linkers with the B_3_N_3_ linkers. Additionally, a valence band (VB) edge at −1.39 V was reported for COF‐CC‐2Ph, proposing an electronic band gap of 3.01 eV [24]. This value should be taken with care, as the VB identification in COF‐CC‐2Ph by STS seems ambiguous, with no obvious spectral differences between COF and pore areas [24]. To probe the VB and to address this issue, we performed UPS measurements of COF‐BN‐2Ph and COF‐CC‐2Ph on Ag(111). The multi‐domain scenario (Figure S6) prevents us from extracting the band structure, but angle‐resolved UPS accurately yields the energy positions of the different VBs. Figure 5 shows the respective energy distribution curves (EDCs) extracted from photoemission intensity maps at 1.45 Å^−1^ (see Figure S17 and Methods Section for more details). For COF‐BN‐2Ph, a clear spectral signature is observed with maximum intensity at an energy of ≈3.4 eV below the Fermi level (*E_F_*). For COF‐CC‐2Ph, two maxima at ≈2.5 and ≈3.4 eV below *E_F_* are discernible. With no contribution for bare Ag(111) in this spectral range, we attribute the signal to the VB of COF‐BN‐2Ph and the VB and VB‐1 of COF‐CC‐2Ph, respectively. This yields an electronic band gap (peak‐to‐peak) of about 5.2 eV for COF‐BN‐2Ph, which is substantially larger than the one of COF‐CC‐2Ph determined by STS only (see above) or by combining our UPS data of the VB with the published value of CB determined by STS (4.12 eV). In any case, our experiments demonstrate that the B_3_N_3_ substitution in the COF induces a renormalization of the band gap. An even larger band gap of 5.79 eV was reported for a quasi‐freestanding boroxine‐linked COF‐BO‐2Ph [22].

**FIGURE 6:**
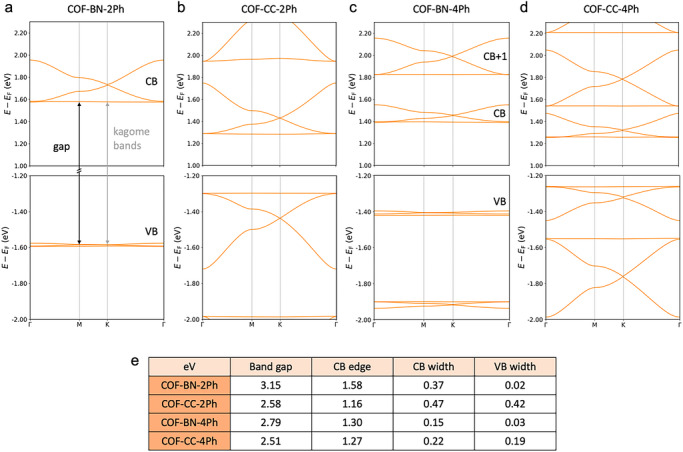
DFT‐calculated electronic structures, along high‐symmetry lines of the 2D hexagonal Brillouin zone. (a–d) Band structure for the four freestanding COF systems, highlighting the conduction (top) and valence bands regions (bottom). The abbreviations CB and CB+1 mark the conductions bands (compare Figures 3 and 4). VB stands for valence band. (e) Table including the calculated values characterizing the band structures of the four systems (compare Figure S19).

To get additional insights on the effect of BN‐substitution and spacer length on the COFs' electronic structure, we performed DFT‐based band structure calculations (see Methods section in the Supporting Information) on four distinct COFs (i.e., COF‐BN‐2Ph, COF‐CC‐2Ph, COF‐BN‐4Ph, COF‐CC‐4Ph) in planar and tilted configurations. The results, presented in Figure 6 and Figure S18, help to rationalize the experimental observations and provide valuable additional insight elusive to experiments, such as the influence of phenyl tilt in the bi/quaterphenyl bridges. Figure 6 shows details of the band structures of the two BN‐substituted systems, i.e., COF‐BN‐2Ph (Figure 6a) and COF‐BN‐4Ph (Figure 6c), together with the two respective all‐C systems, COF‐CC‐2Ph (Figure 6b) and COF‐CC‐4Ph (Figure 6d). In all cases, the CB and VB show a kagome‐type structure, i.e., a flat band combined with more dispersive bands forming a Dirac cone [58]. Band structure plots with continuous, extended energy scales and corresponding total density of states (TDOS) are shown in Figure S18. With the strong DOS contribution of the first CB band on the spacer units (see Figures 3d and 4d), the kagome‐type bands can be related to the kagome lattice of the network (see Figure 1). Local density of states (LDOS) calculations confirm this interpretation (right panels in Figures 3c and 4c). The LDOS plots represent an integration over the CBs and CB+1, respectively. Considering the simplicity of the model (free‐standing COF, fixed phenyl dihedral angles), the agreement with the experimental dI/dV maps (middle panels in Figures 3c and 4c) is satisfactory. Specifically, the spectral weight of the CB on the carbon backbone, the modified symmetry when proceeding from the CB to CB+1 for COF‐BN‐4Ph, and the negligible contribution of the B_3_N_3_ nodes are captured. The identification of kagome bands and the definition of CB and VB are fully consistent with findings reported for the boroxine‐linked COF‐BO‐2Ph [22]. The experimental dI/dV spectra (Figures 3b and 4b) do not resolve the signatures of the flat and dispersing bands individually, due to broadening effects on the metallic support [22]. Kagome band structures in different π‐bands were theoretically identified in a series of COF structures, including COF‐BS‐1Ph [49] and COF‐CC‐2Ph [12], and attract considerable interest for the design of quantum materials [12, 44, 49, 59, 60]. To ease the comparison between the four systems, four parameters extracted from the band structure calculations are listed in the table in Figure 6e, namely electronic band gap, CB edge, CB, and VB width (for additional values, see Figure S19). The main experimental findings are corroborated by the calculations that do not consider the metallic support. Namely, an upshift of the CB edge when substituting CC by BN, accompanied by a widening of the band gap, a downshift of the CB edge in the BN‐substituted COFs when increasing the spacer length (from 2Ph to 4Ph), and a concomitant decrease of the CB band width are observed (Figure 6). Specifically, the last effect is visualized in the experimental voltage‐dependent dI/dV maps (Figures S12 and S15). For COF‐BN‐2Ph, only the first CB band is probed within 0.6 V. For COF‐BN‐4Ph, both the CB and CB+1 contribute due to their narrower band widths. Importantly, the calculations reveal an increase of the band gap for the B_3_N_3_‐linked COFs compared to the benzene‐linked ones, which is expected due to the polar/more insulating nature of the linker. Note that the absolute values of the calculated band gaps are not compared to experiments due to the well‐known shortcomings of DFT in precisely predicting band gaps [61, 62]. The electron‐hole symmetry apparent for the (planar) all‐C COFs is broken by introducing the B_3_N_3_‐nodes, which induce a CB width clearly exceeding the VB width. Furthermore, the calculations reveal a band gap reduction for hypothetical, fully planar COFs, as compared to the models with tilted phenyl rings in the backbones (Figure S19).

The COF structure and electronic properties can be tuned at the atomic scale by applying site‐selective dehydrogenation. Specific tunneling conditions and in particular, bias voltages exceeding 2.1 V induce irreversible modifications in the STM appearance of the COF arrays. These changes can be realized locally by recording tunneling spectra, by applying voltage pulses, or by scanning at 2.5 V. The contrast modifications are apparent in STM data (see Figure S20), with a dark appearance and symmetry reduction of B_3_N_3_ nodes, disrupting the homogeneous honeycomb‐like contrast of the COF array. Not only the nodes, but also the adjacent sections of the quaterphenyl bridges show a reduced apparent height, pointing to structural distortions, i.e., a bending toward the substrate. We attribute these effects to the dehydrogenation of N sites of the B_3_N_3_ linkers [63]. For individual borazine (B_3_N_3_H_6_) units on Ag(111), dehydrogenation by molecular manipulation was reported to occur at B sites [64]. Nc‐AFM was used to substantiate the claim of site‐selective dehydrogenation and to explore concomitant structural changes (Figures S21 and S22). Spatially‐resolved STS shows an upshift in energy of the CB feature upon dehydrogenation of B_3_N_3_ nodes (Figure S20f).

## 3 Conclusion

We have synthesized unprecedented single‐layer BN‐substituted COFs with two distinct periodicities and pore sizes, namely COF‐BN‐2Ph and COF‐BN‐4Ph, on Ag(111) and Au(111). Bond‐resolved AFM reveals a non‐planar configuration of the bi/quaterphenyl bridges. The COF's electronic structure was characterized by STS, dI/dV mapping, and DFT calculations. The experiments evidence that longer bridging units decrease both the conduction band energy and width. BN substitution induces an increase in the band gap and a reduction of the frontier band dispersions, which can be tuned with the spacer length. Specifically, combined UPS and STS data reveal a gap of 5.2 eV for COF‐BN‐2Ph, clearly exceeding the gap of 4.12 eV of COF‐CC‐2Ph. Accordingly, beyond adding B_3_N_3_ linked single‐layer COFs to the family of covalent architectures on surfaces characterized on the atomic level, we demonstrated how BN‐substitution, as well as spacer length and planarity, represent control knobs to tune the electronic structure of COFs. Our findings thus provide crucial insights for developing the next generation of functional 2D COFs.

## Conflicts of Interest

The authors declare no conflict of interest.

## Supporting information




**Supporting File**: smll73718‐sup‐0001‐SuppMat.pdf.

## Data Availability

The data that support the findings of this study are available from the corresponding author upon reasonable request.
