# Supplementary material for: On‐Surface Synthesis of B3N3‐Substituted Two‐Dimensional Covalent Organic Frameworks with Distinct Pore Sizes and Kagome Band Structures
Source: Small. 2026 May 22;22(32):e73718. doi: 10.1002/smll.73718 (PMC13244414; doi:10.1002/smll.73718)
Supplement: Supplementary file 1 — Supporting File: smll73718‐sup‐0001‐SuppMat.pdf. [file SMLL-22-e73718-s001.pdf]

SUPPORTING INFORMATION

On-Surface Synthesis of B_3N_3-substituted Two-Dimensional Covalent Organic Frameworks with Distinct Pore Sizes and Kagome Band Structures

Birce Sena Tömekçe,[1] Alireza Nazari Khodadadi,[2] Laura Caputo,[3,4] Ignacio Piquero-Zulaica,*[1,5,6] Martina Corso,[5] Frederik Schiller,[5] J. Enrique Ortega,[5,7] Jean-Christophe Charlier,*[3] Luigi Vaccaro,*[2] and Willi Auwärter*[1]

[1] Physics Department E20, TUM School of Natural Sciences, Technical University of Munich, Garching, Germany
[2] Laboratory of Green S.O.C. – Dipartimento di Chimica, Biologia e Biotecnologie, Università degli Studi di Perugia, Perugia, Italy.
[3] Institute of Condensed Matter and Nanosciences, Université catholique de Louvain (UCLouvain), Louvain-la-Neuve, Belgium
[4] Department of Chemical Sciences, University of Naples 'Federico II', Napoli, Italy
[5] Centro de Física de Materiales (CSIC-UPV/EHU)- Materials Physics Center, San Sebastian, Spain
[6] IKERBASQUE, Basque Foundation for Science, Bilbao, Spain
[7] Departamento de Física Aplicada, Universidad del País Vasco, San Sebastian, Spain

Table of Contents

- **Methods (STM, AFM, UPS, DFT)** Page 2 - 3
- **Additional Data (STM, AFM, UPS, DFT)**

  Figures S1 - S13 Page 3 - 11

  Surface State Confinement Page 12

  Figures S14 - S19 Page 12 -15

  Tip Induced Dehydrogenation Page 16 -19

  Figures S20 - S22 Page 17 -19

- **Synthesis of TPB and TBB** Page 20 -23
- **References** Page 24

Methods

Scanning probe microscopy. The experiments were performed in two different UHV setups, one equipped with Createc low-temperature STM operating at 6.5 K and the other with a Createc STM/AFM instrument having a qplus sensor which operates at 5 K. In both setups, the samples were prepared in-situ. The single crystals were cleaned by sputtering (Ar^+ ions at an energy of 1 keV) and annealing (at ~490 °C) cycles, prior to precursor deposition. TPB and TBB were deposited from Knudsen cells by using a Dodecon organic molecular beam epitaxy (OMBE) evaporator at 220 °C and 260 °C, respectively in the LT-STM chamber. In the STM/AFM chamber, a homemade OMBE was used to deposit TPB (at 210 °C) and TBB (at 280 °C). STM images were acquired in constant current mode by applying a bias voltage to the sample, whereas AFM measurements were performed at constant height mode (open Z-feedback) by applying 0 bias voltage and modulating the frequency of the qplus sensor (oscillation amplitude: 60 pm, resonance frequency: ~30 kHz, Q-factor: ~45000). The images were processed using WSxM software.[1] dI/dV spectroscopy in both systems was performed with open feedback loop using an internal lock-in amplifier (modulation frequency: 403 Hz, modulation amplitude: 30-40 mV, see figure captions).

UPS. TPB and the all-C reference were deposited on Ag(111) with the sample at 100 °C. Then the sample was annealed slowly to 200 °C and finally to 230 °C - 260 ºC (Br atoms should still be present on the surface). UPS measurements were performed in UHV (base pressure of 1 × 10^{-10} mbar) with a display-type hemispherical electron analyzer (SPECS Phoibos 150), an energy/angle resolution of 40 meV per 0.1° and a monochromatized Helium I ($h\nu$= 21.2 eV) source. The sample temperature during UPS measurements was set to RT. Angle-resolved UPS (E-E_F vs k_y) measurements were performed along the $\overline{\Gamma K}$ direction of Ag(111). The EDCs were extracted at k_y = 1.45 Å^{-1}.

DFT. All calculations were performed using DFT with norm-conserving pseudopotentials from the PseudoDojo library [2] and a plane-wave basis set as implemented in the Quantum ESPRESSO package.[3] The exchange-correlation energy was treated within the generalized gradient approximation (GGA) using the Perdew–Burke–Ernzerhof (PBE) functional.[4] An energy cutoff of 85 Ry was used for the plane-wave expansion, and the Brillouin zone was sampled using a 12×12×1 Monkhorst-Pack k-point mesh to compute the Density of States (DOS) and the band structures. After full relaxation of the unit cell parameters, the tilt angles

of the phenyl rings were manually adjusted to match experimental observations, in order to better reproduce the corresponding electronic band structures.

LDOS plots have been computed using the Quantum ESPRESSO code [3] and the Critic2 software [5,6] using PAW pseudopotentials to obtain clear images.[7] All plots have been computed using a constant height mode, with a distance of 4.5 Angstrom above the last atom before the vacuum along the z direction. Moreover, a 4x4x1 supercell has been considered for the LDOS plotting.

AI tools. No content was created using AI tools. However, such tools were used on selected sentences written by the authors to check grammar and improve linguistic clarity.

Additional Data (STM, AFM, UPS, DFT)

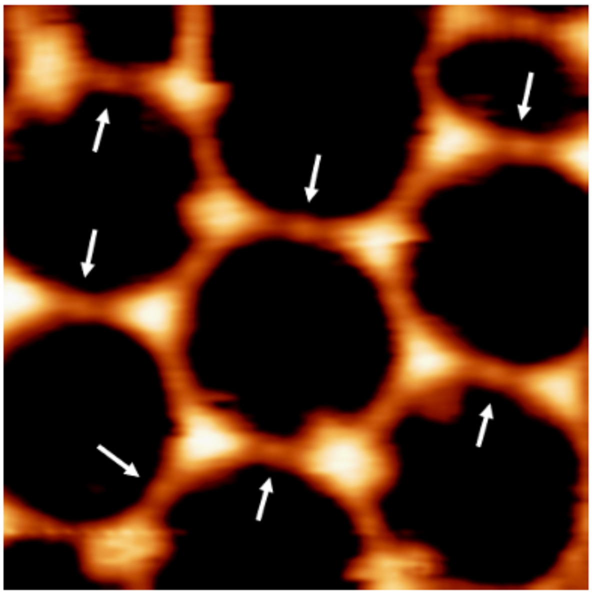

Figure S1: The STM image of the organometallic network formed by TBB on Ag(111) at 260°C. Ag atoms engaged in organometallic bonding are indicated by arrows. (Image size: 10 x 10 nm, I: 150 pA, V_b: 0.5 V).

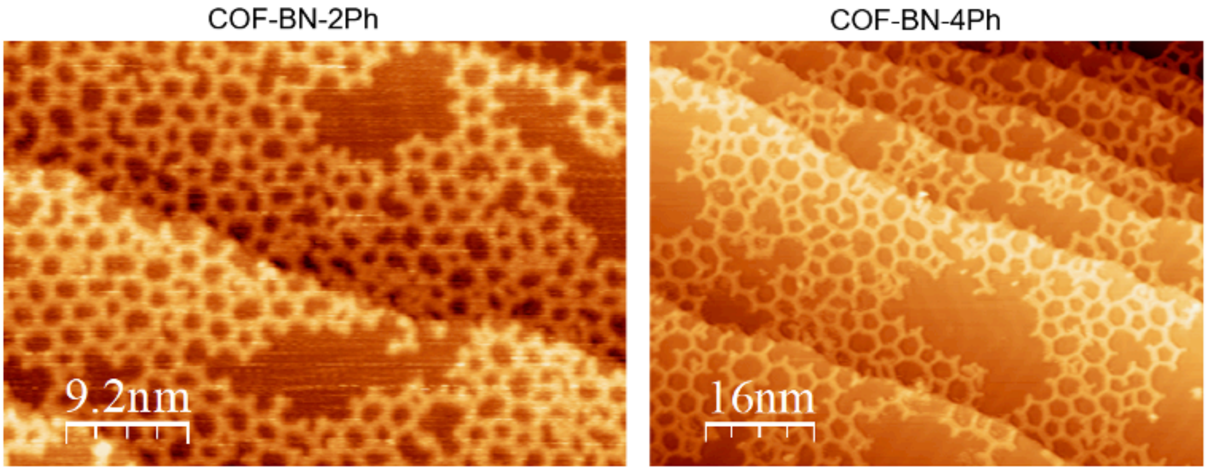

Figure S2: The large-scale STM images showing COF-BN-2Ph and COF-BN-4Ph domains on Au(111). (I: 150 pA, V_b: 1 V and I: 200 pA, V_b: 0.5 V, respectively).

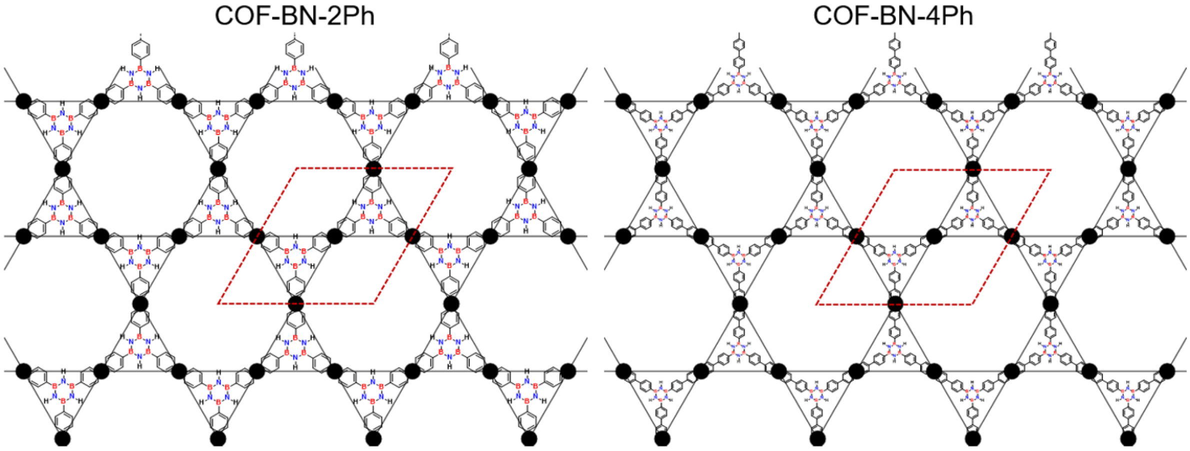

Figure S3: Kagome lattice overlay on the spacer units of COF-BN-2Ph and COF-BN-4Ph structures. The rhombic unit cell is indicated on both COFs. The calculated dimensions are 2.31 nm for COF-BN-2Ph, 3.82 nm for COF-BN-4Ph, 2.26 nm for COF-CC-2Ph, and 3.77 nm for COF-CC-4Ph.

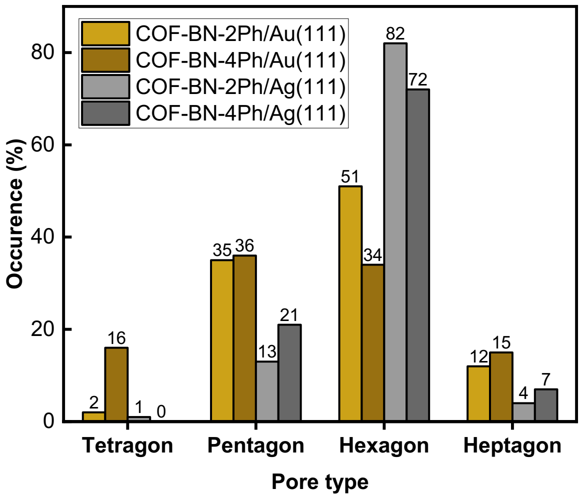

Figure S4: Comparative statistical analysis of the pore types constituting the COFs grown on Au(111) and Ag(111). Rarely observed octagons and distorted, open pores were excluded. The best structural quality, i.e. the largest percentage of hexagons was obtained on Ag(111). On both substrates the percentage of hexagons in COF-BN-2Ph exceeds that of COF-BN-4Ph, in line with the reduced flexibility of the precursor. The wider distribution of pore types on Au(111), particularly for COF-BN-4Ph, indicates a lower structural quality. For the statistical analysis, large scale STM images with a clear resolution were considered. The total numbers of pores (tetragons, pentagons, hexagons and heptagons) counted for COF-BN-2Ph/Au(111), COF-BN-4Ph/Au(111), COF-BN-2Ph/Ag(111), and COF-BN-4Ph/Ag(111) are 137, 89, 211, and 149, respectively.

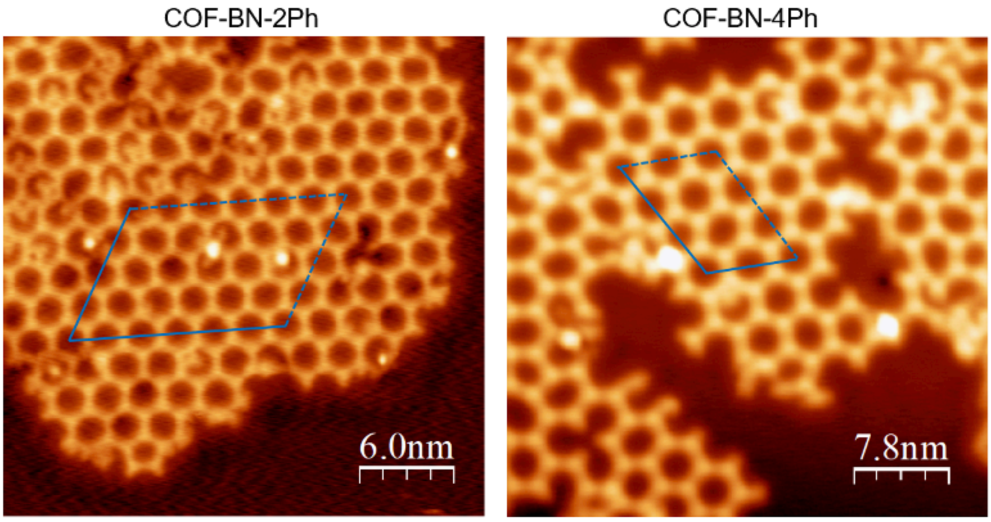

Figure S5: COF islands with large crystalline domains on Ag(111). For COF-BN-2Ph, a domain with 5 x 7 hexagons, for COF-BN-4Ph a domain with 3 x 4 hexagons is shown. (COF-BN-2Ph, I: 10 pA, V_b: 0.02 V; COF-BN-4Ph I: 10 pA, V_b: 0.2 V)

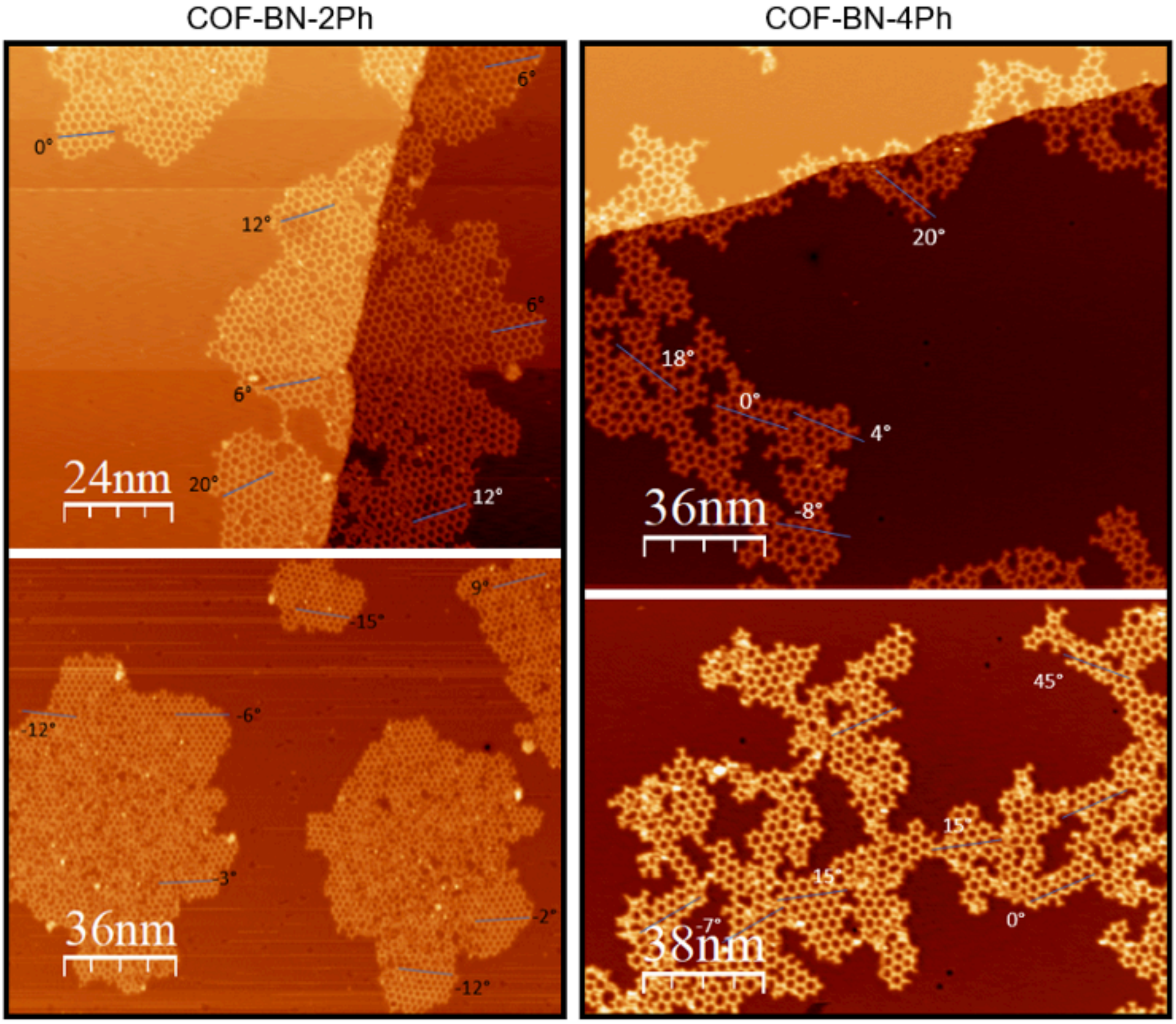

Figure S6: Large scale STM images of COF-BN-2Ph and COF-BN-4Ph islands on Ag(111), showing the network characteristics. The angles of rotational domains are indicated, with the angle of 0° serving as a reference (defined independently for both COFs). A variety of coexisting domain orientations is observed, which do not follow an integer multiple of one specific angle. (Imaging parameters: 20 pA, 0.1 V (upper), 40 pA, 0.1 V (lower) for COF-BN-2Ph; 20 pA, 0.2 V for both COF-BN-2Ph images.)

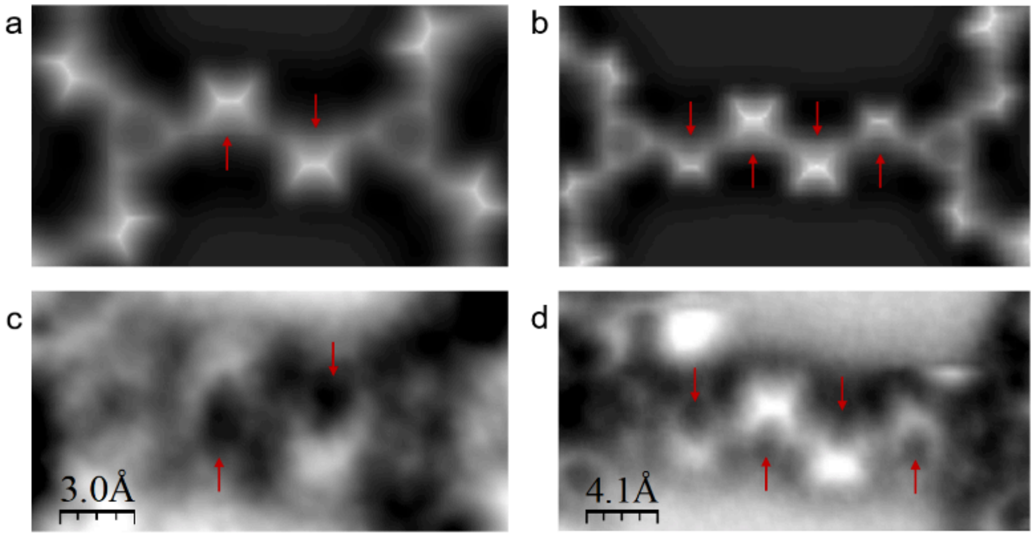

Figure S7: Estimation of phenyl ring dihedral angles. Probe particle model simulations of the structural models of (a) COF-BN-2Ph with 15° phenyl twist angle, (b) COF-BN-4Ph with the middle phenyl angles of 20°, phenyls linked to B_3N_3 units 10°.[8,9] (c) and (d) Close-up Δf images of COF-BN-2Ph and COF-BN-4Ph on Ag(111), displaying twisted geometry of the phenyl rings. Larger tilt angle of the middle phenyls compared to the B_3N_3-linked phenyls is visible.

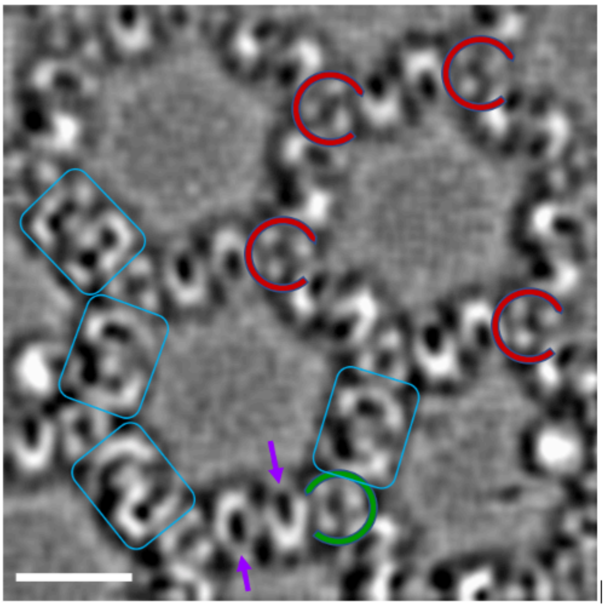

Figure S8: Laplace filtered Δf image of COF-BN-2Ph on Ag(111), illustrating the chirality induced by the tilt direction of the phenyl rings. Clock-wise chirality of phenyl rings bound to B_3N_3 linkers is marked by red arrows, counter clock-wise chirality by a green arrow. Purple arrows indicate biphenyl rings introducing a counter clock-wise character. For some biphenyl spacers (highlighted in blue) the tilt direction cannot be clearly determined. (Scale bar: 1nm).

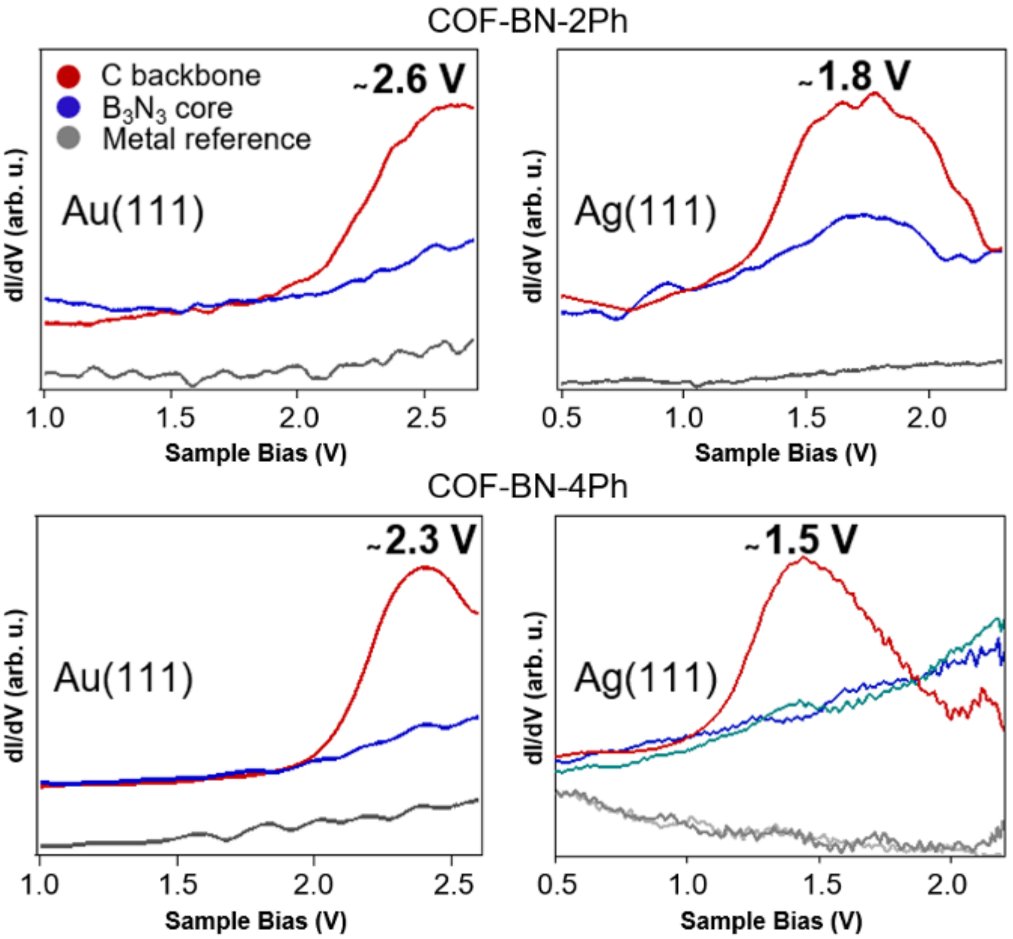

Figure S9: Point STS curves taken on COF-BN-2Ph and COF-BN-4Ph on Au(111) and Ag(111). The CB state is detected as a broad peak on biphenyl or quaterphenyl segments, with a smaller FWHM and a lower voltage onset (0.3 V) in the case of COF-BN-4Ph, consistently on both substrates. (Tip stabilization parameters: COF-2Ph/Au(111): 150 pA, 2.7 V, COF-2Ph/Ag(111): 100 pA, 2.3 V, COF-4Ph/Au(111): 200 pA, 2.6 V, COF-4Ph/Ag(111): 200 pA, 2.2 V).

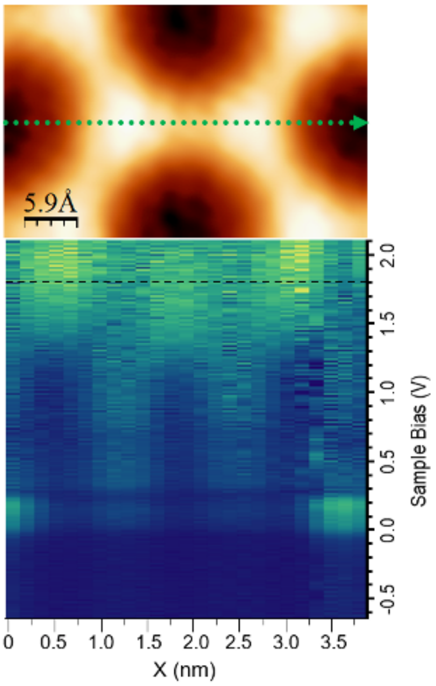

Figure S10: Line STS map measured on COF-BN-2Ph on Ag(111), similar to Figure 3a, but representing a different area. In this case, the first and last CB lobes appear symmetrical. (Tip stabilization parameters: 80 pA, 2.1 V).

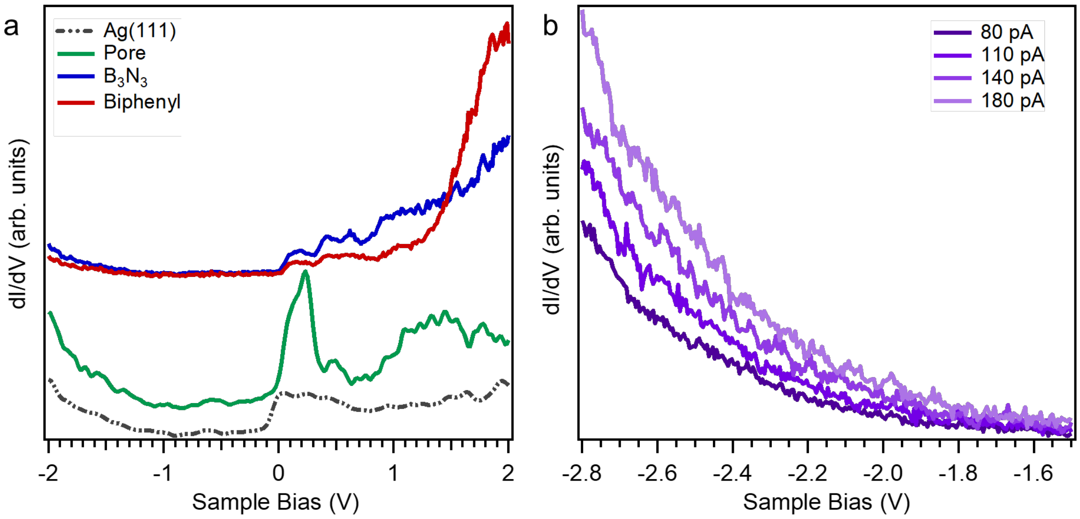

Figure S11: dI/dV spectra taken on COF-BN-2Ph on Ag(111). (a) Point STS curves symmetric around the Fermi level, acquired on different positions (Tip stabilization parameters: 90 pA, 2.0 V, lock-in modulation amplitude: 30 mV). (b) STS curves acquired on biphenyl bridge with increasing tunneling current setpoints, representing the occupied states regime. (Tip stabilization voltage: -1.5 V, lock-in modulation amplitude: 40 mV).

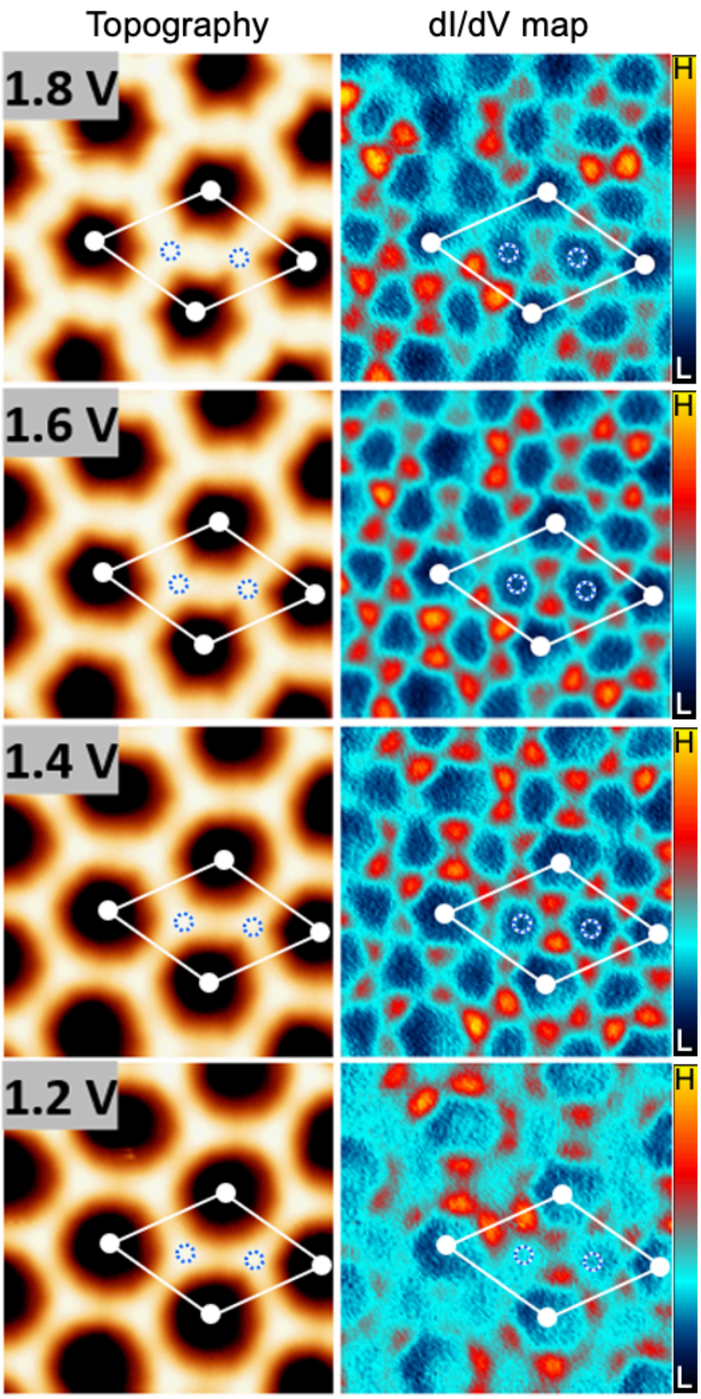

Figure S12: Series of voltage-dependent STM images (left column) and corresponding, simultaneously recorded constant current dI/dV maps (right column). The white dots mark pore centers, the rhombi outline the COF unit cell and the blue dashed circles highlight positions of the B_3N_3 nodes. (Image size: 6.0 nm x 6.0 nm, I= 180 pA). In addition to the "fading-out" of the dumbbell contrast at 1.2 V, due to a minor contribution of the CB at this energy, a pronounced variation of the dumbbell contrast both within each dI/dV map and between maps is observed. These inhomogeneities are ascribed to subtle structural differences in the spacer units, which can arise from a variation in the phenyl tilt angles and/or directions (see Figure

S8). The slight asymmetry in the STS map shown in Figure 3a, with the three CB lobes appearing at different energies, is attributed to the same effect. Additional line STS measured from different pores yield fully symmetric STS maps (Figure S10).

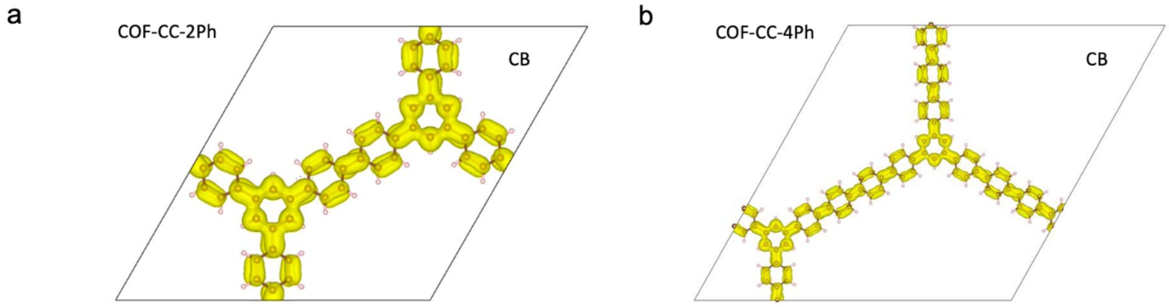

Figure S13: DOS plots for the COF-CC-2Ph (a) and COF-CC-4Ph (b) unit cells representing the CB (compare Figure 3d and Figure 4d, respectively). The isosurfaces represent a value of 0.05 states/Bohr^3*Ry. The spatial distribution of the calculated density of states for the first CB of COF-CC-2Ph and COF-CC-4Ph shows considerable weight on the benzene linkers. This contribution is clearly more extended compared to the BN-substituted analogues that reveal a reduced weight on the B_3N_3 nodes (compare Figure 3d and Figure S16).

Surface State Confinement The STS data for COF-BN-2Ph and COF-BN-4Ph on Ag(111) reveal the influence of the COFs on the surface electronic structure of Ag(111), specifically highlighting confinement effects in the pores exposing bare Ag(111). For COF-BN-2Ph, the first confined state (n=1) is located at ~170 meV (compare Figure 3a and b). On bare Ag(111), the surface state has an onset at around -67 meV.[10] For COF-BN-4Ph, the n=1 confined state is located at 20 meV, reflecting the larger pore size compared to COF-BN-2Ph.[11] Figure S14 summarizes the surface state confinement in COF-BN-4PhAg(111). The effect of site-selective dehydrogenation of the B_3N_3 nodes on the surface state confinement is discussed in Figure S20g.

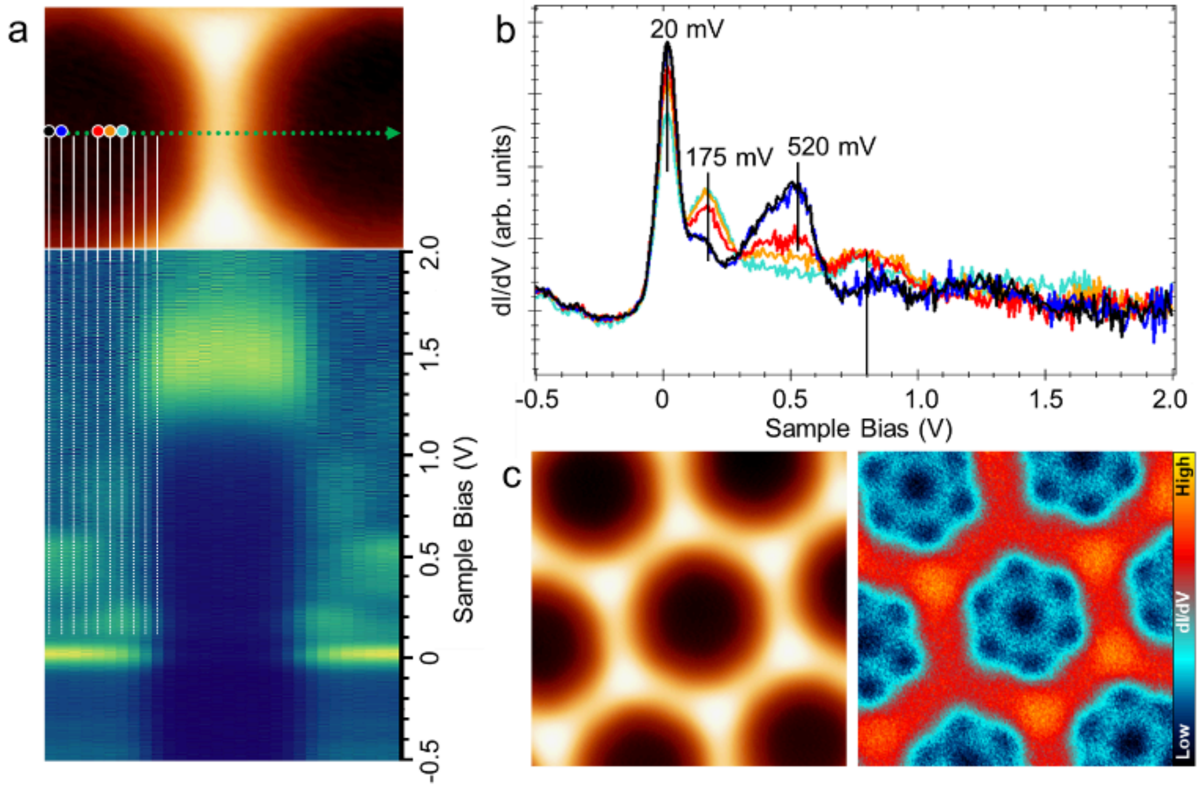

Figure S14: Surface state confinement in the pores of COF-BN-4Ph on Ag(111). (a) STS map showing the spectra measured along the line in the STM image on top, from pore center to pore center. (Image size: 3.7 x 3.2 nm, I = 100 pA, V = 0.1 V), (Tip stabilization parameters: 100 pA, 2 V). (b) Selected point spectra from the map highlight intensity variations of the resonant states over distinct positions, namely pore center and half-way. Each spectrum is marked by corresponding colors in the topography image and indicated in the map. The prominent peak at 20 mV represents n=1, the resonance at 175 mV n=2 and the broad signature at 520 mV is assigned to n=4 state. (c) Topography (left) and the corresponding dI/dV map (right) acquired at 0.8 V, showing the spatial distribution of the higher states. (Image size: 7.0 x 7.0 nm, I: 180 pA).

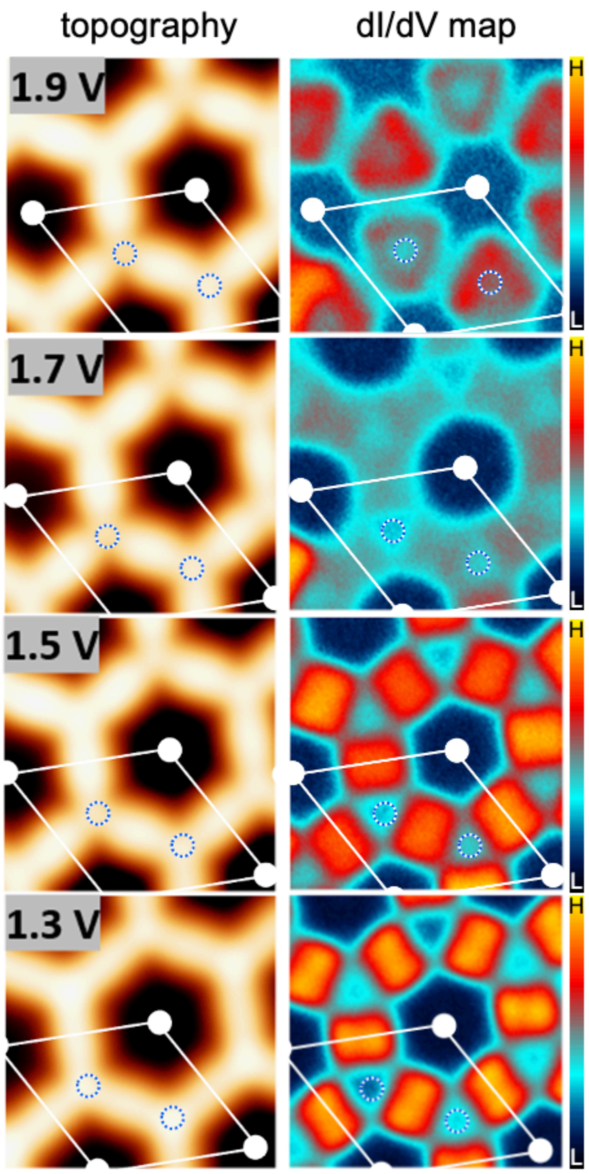

Figure S15: Series of voltage-dependent STM images (left column) and corresponding, simultaneously recorded constant current dI/dV maps (right column). The white dots mark pore centers, the rhombi outline the COF unit cell and the blue dashed circles highlight positions of the B_3N_3 nodes. (Image size: 6.0 nm x 6.0 nm, I= 180 pA; Linear color scales in arb. units, H and L mark high and low intensity, respectively).

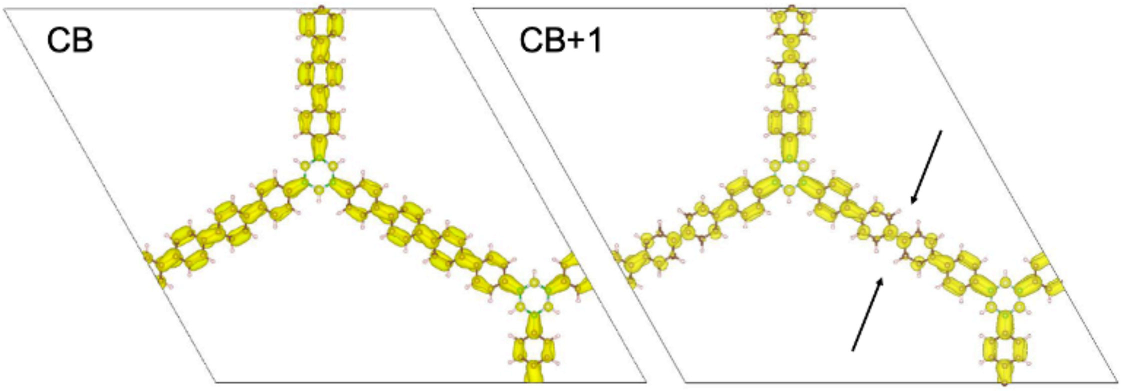

Figure S16: Enlarged version of Figure 4 (d), showing the DOS plot for free-standing COF-BN-4Ph unit cell representing the first CB (left panel) and the second CB (right panel, CB+1).

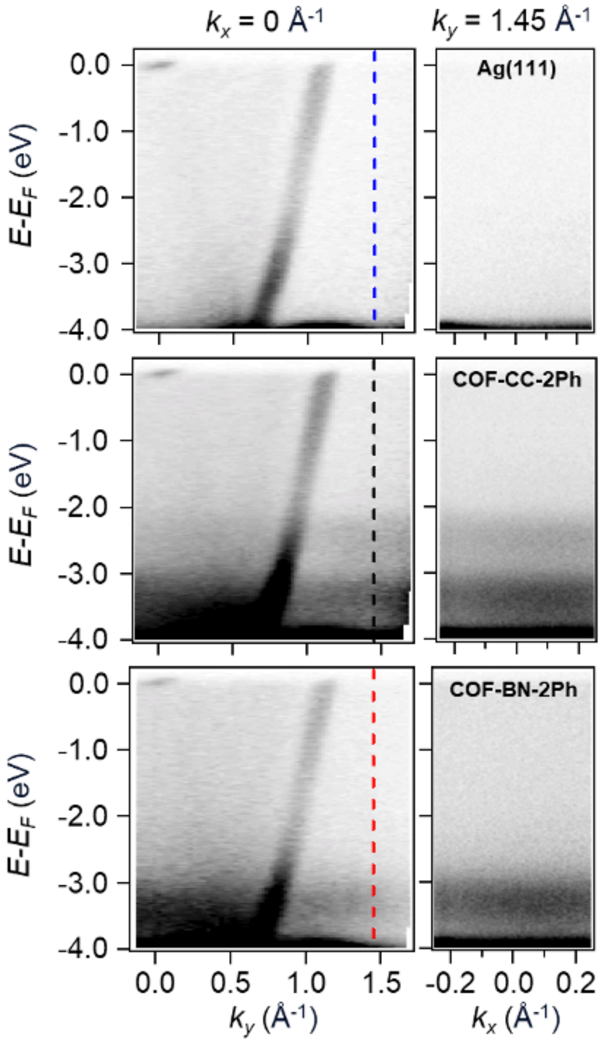

Figure S17: Angle-resolved UPS photoemission intensity maps (E-E_F vs k_y and E-E_F vs k_x plots) of pristine Ag(111), COF-CC-2Ph/Ag(111) and COF-BN-2Ph/Ag(111). E-E_F vs k_y maps

are taken along the $\overline{\Gamma K}$ direction of Ag(111). The EDCs presented in the main text represent the data extracted along the dashed lines (at 1.45 $Å^{-1}$). The highly dispersive band appearing in all three systems corresponds to Ag-sp bulk bands. The intensity close to E_F at Γ corresponds to the Ag surface state.

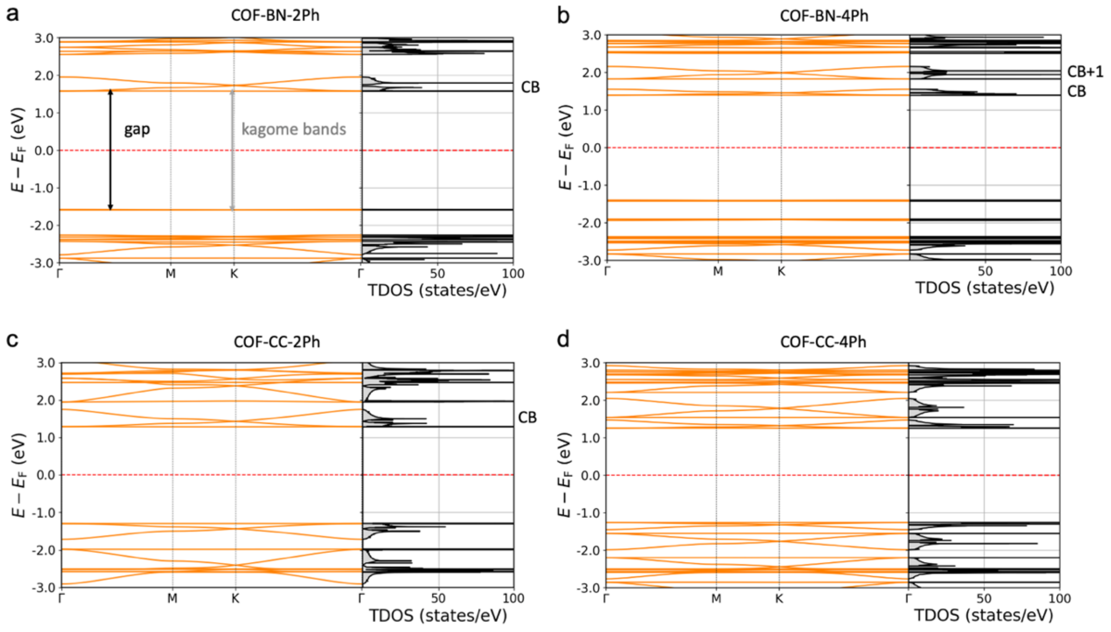

Figure S18: DFT-calculated electronic band structures and corresponding total density of states (TDOS) of the two BN-substituted systems, *i.e.*, COF-BN-2Ph (a) and COF-BN-4Ph (b), together with the two respective all-C systems, COF-CC-2Ph (c) and COF-CC-4Ph (d). The abbreviations CB and CB+1 mark the conductions bands (compare Figure 3 and 4). The band structure shown in (c) matches literature reports for COF-CC-2Ph.[12] Key parameters describing the calculated band structures of all systems are provided in the table in Figure S19.

all in eV	Band Gap	CB edge	CB width
Tilted COF-CC-2Ph	2.57	1.30	0.46
Tilted COF-BN-2Ph	3.01	1.53	0.43
Tilted COF-CC-4Ph	2.45	1.23	0.23
Tilted COF-BN-4Ph	2.76	1.40	0.17
Flat COF-CC-2Ph	2.34	1.22	0.42
Flat COF-BN-2Ph	2.92	1.49	0.36
Flat COF-CC-4Ph	2.15	1.10	0.17
Flat COF-BN-4Ph	2.36	1.20	0.14

Figure S19: Table comparing the values obtained from the calculated band structures of all the systems including tilted and flat phenyl configurations. Note that the values for the tilted configurations are slightly different compared to Figure 6, as a different calculations scheme was applied for the comparison provided here (VASP versus Quantum ESPRESSO).

Tip-induced dehydrogenation. Nc-AFM was used to substantiate the claim of dehydrogenation and to explore concomitant structural changes. To this end, an area of a COF-BN-4Ph array that includes both intact and dehydrogenated sites (see STM image in Figure S20a) is scanned by nc-AFM at different tip heights (Figure S20b, c and d). The pore that is completely contained in the Δf image in Figure S20b features three intact B_3N_3 linkers indicated by blue circles in the corresponding Laplace-filtered image (Figure S20d). These nodes show the characteristic three-lobe structure, with N-H sites appearing as protrusions (compare Figure 2). All other B_3N_3 linkers in the imaged area have at least one dehydrogenated N site, identified by a missing protrusion (highlighted by red arrows). Close-up images of the B_3N_3 units taken from Figure S20c and S20d are provided in Figure S21 to highlight the differences. The difference between intact and dehydrogenated B_3N_3 units gets more pronounced in Δf images recorded at smaller tip-sample distance (Figure S20c). Here, intact B_3N_3 units show a ring-like symmetry whereas partially dehydrogenated B_3N_3 units feature individual, pronounced protrusions reflecting N-H sites and "missing" protrusions for dehydrogenated N sites. The respective unfiltered Δf image is presented in Figure S22 jointly with data recorded at even smaller tip sample distance that confirm the missing contributions from dehydrogenated N sites and furthermore reveal a triangular appearance of intact B_3N_3, now dominated by the B sites. No triply-dehydrogenated nodes could be identified in the data, although such complete dehydrogenation cannot be ruled out in principle. Phenyl rings adjacent to (partially) dehydrogenated B_3N_3 nodes appear darker and less tilted compared to the rings adjacent to intact nodes (Figures S20b-d). In contrast, the central two phenyl rings in the quaterphenyl bridges are not obviously affected by the nodal structure. Accordingly, we conclude that dehydrogenation increases the interaction of the B_3N_3 units with the Ag(111) surface, reducing the adsorption height and pulling the attached phenyl rings closer to the surface. Such a distortion is consistent with the STM appearance (see Figure S20a, e).

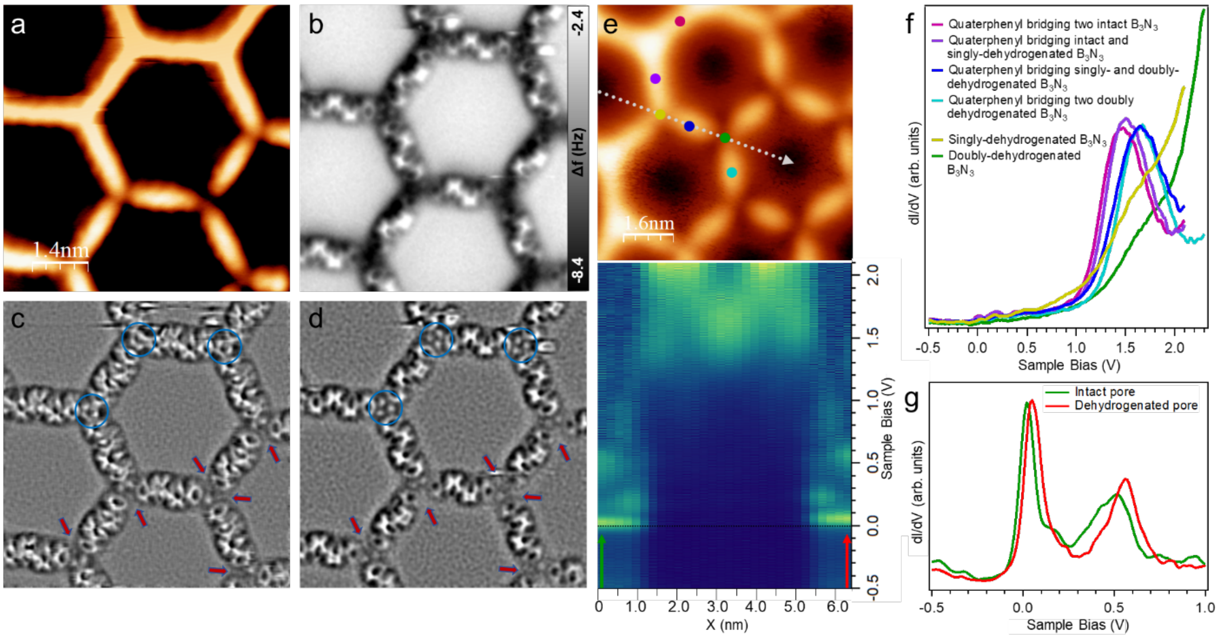

Figure S20. Site-selective dehydrogenation of COF-BN-4Ph with modification of the electronic structure. (a) STM image of the area probed by nc-AFM (b-d) including intact and dehydrogenated nodes. Dehydrogenation was induced by positioning the tip on B_3N_3 nodes in tunneling with parameters: 2.7 V and 50 pA. (Imaging parameters: 30 pA, 20 mV.) (b) Δf image and (d) corresponding Laplace filtered image. The blue circles highlight intact B_3N_3 nodes. Dehydrogenated N sites are marked by arrows. (c) Laplace filtered Δf image recorded at reduced tip sample distance (40 pm). The unfiltered image is provided in Figure S15a. (e) STS map representing spectra recorded along the dashed line in the STM image on top. Dehydrogenation was induced by scanning the area at 100 pA and 2.5 V. The colored dots in the top panel mark the positions of the spectra presented in (f) and the green and red arrows in the bottom panel highlight the spectra compared in (g). (Imaging parameters: 30 pA, 0.3 V. Stabilization parameters: 90 pA, 2.1 V, lock-in modulation amplitude: 40 mV.) (f) Individual spectra revealing an upshift of the CB peak upon dehydrogenation of adjacent nodes (Stabilization parameters: 90 pA, for green and cyan curves at 2.3 V, for the other curves at 2.1 to avoid dehydrogenation, lock-in modulation amplitude: 30 mV). (g) Spectra recorded at the centers of a pristine and a "dehydrogenated" pore.

Dehydrogenation thus can be a strategy to tune the electronic structure by planarization of the phenyl backbones as calculated in Fig. S19. Site-selective dehydrogenation of N and C sites by STM was previously demonstrated for graphene nanoribbons and individual molecules on

coinage metal supports, typically using voltage pulses of 3 V or higher,[13,14] thus exceeding the threshold applied in the present case. For example, dehydrogenation of a tripyrrin derivative on Ag(111) induced a depression in STM images and a loss of atomic contrast in nc-AFM data.[15] Similarly, graphitic N at graphene nanoribbon edges was reported to appear dark in nc-AFM images and a lower adsorption height was shown by DFT.[16] These observations are reminiscent of the findings described in Figure S20a-d.

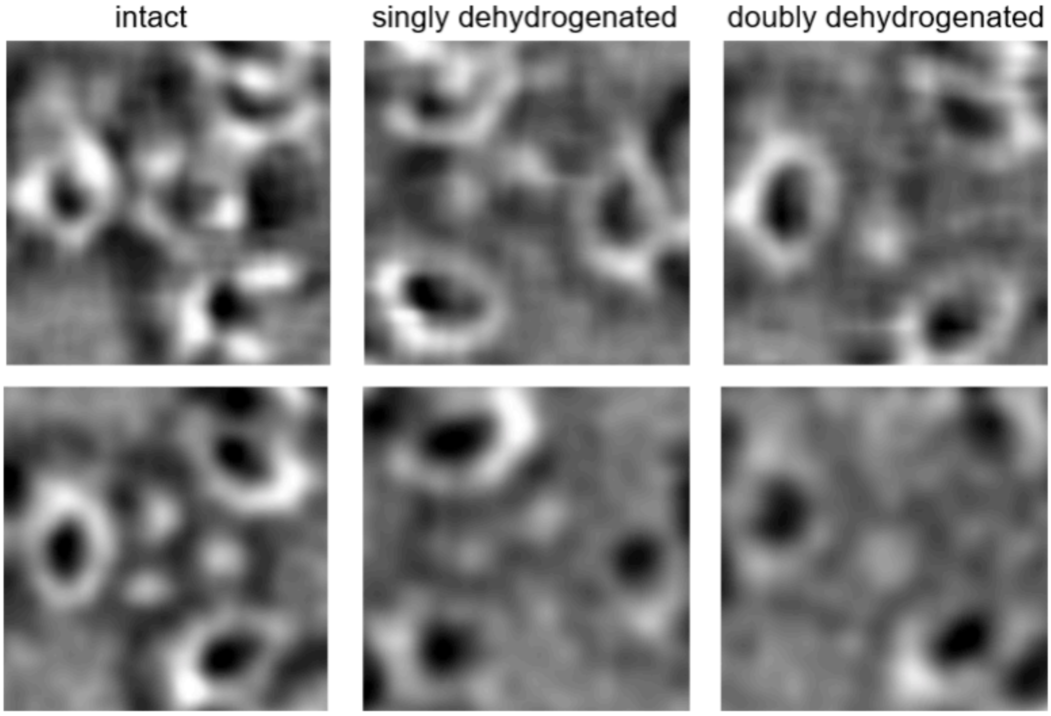

Figure S21: Close-up Laplace filtered, Δf images of the intact, singly and doubly dehydrogenated B_3N_3 nodes, taken from Figure S20c (upper row) and d (lower row). The bright protrusions are missing on the dehydrogenated sites, exhibiting the same contrast as the surface. Note that the missing protrusions can be distinguished easier from the images taken at closer z setpoint (upper row).

The dehydrogenation is reflected in STS data, again recorded on an area including both intact and dehydrogenated B_3N_3 nodes (Figure S20e). The STS map shown in the bottom panel of Figure S20e reflects a path from pore center to pore center, crossing two partially dehydrogenated B_3N_3 nodes and the connecting quaterphenyl bridge (see dashed arrow in the STM image in the top panel). The central lobe in the STS map is clearly upshifted in energy

compared to the CB feature in pristine COF-BN-4Ph (Figure 4a). Individual spectra recorded on characteristic positions of the network, including the quaterphenyl on the line, are shown in Figure S20f and confirm an upshift related to the dehydrogenation of B_3N_3 nodes. The CB peak measured on the segment bridging a singly- and doubly-dehydrogenated B_3N_3 unit (blue marker in Figure S20e) is located at 1.66 V, while the segment bridging two intact B_3N_3 units (pink marker) exhibits a CB peak at the characteristic voltage of ~ 1.5 V. Without a characteristic contribution to STS, the dehydrogenation does not considerably affect the spectra recorded on the different B_3N_3 nodes. Dehydrogenation does not only lead to an upshift of the CB, but also affects the surface state confinement in the pores. In Figure S20g, the spectrum taken at the center of the intact pore (green) is plotted together with the one measured on the partially dehydrogenated pore (red), revealing an upshift of ~ 30 mV for the n=1 confined state with dehydrogenation. The selected spectra are also marked in the STS map by arrows (Figure S20e). The upshift is presumably attributed to an enhanced scattering of surface electrons with the 2D COF scattering barriers.[4]

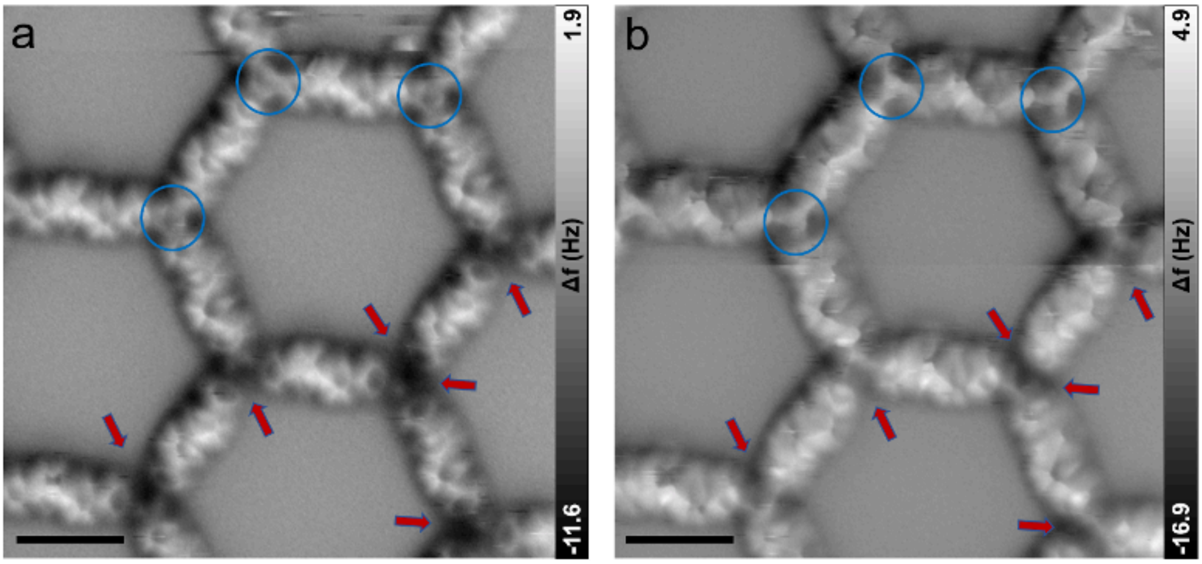

Figure S22: Δf images combining intact, singly and doubly dehydrogenated B_3N_3 nodes, taken from the area shown in Figure S20a-d. (a) corresponds to the Laplace filtered image in Figure S20c. (b) was acquired at 40 pm closer tip-sample distance. Scale bars: 1.4 nm.

SYNTHESIS OF TPB AND TBB

Unless otherwise stated, chemicals were purchased from Merck, TCI, Fluorochem and used as obtained from commercial sources without further purification. The catalyst, $Hf(OTf)_4$, was dried in glass oven (Buchi, B-585) under vacuum for 24h at 120ºC. Melting points were measured on a Büchi 510 apparatus. Nuclear magnetic resonance (NMR) characterizations were recorded on a Bruker DRX-ADVANCE 400 MHz (^{1}H at 400 MHz and ^{13}C at 100.6 MHz) using the solvent residual signal as an internal reference ($CDCl_3$: δH = 7.26 ppm, δC = 77.16 ppm). Chemical shifts are reported in ppm (δ), coupling constant (*J*) in hertz and multiplicity are reported as follows: *s* = singlet, *d* = doublet, and *m* = multiplet. Boronate starting materials (2-(4-bromophenyl)-1,3,2-dioxaborolane and 2-(4'-bromo-[1,1'-biphenyl]-4-yl)-1,3,2-dioxaborolane) were prepared from their corresponding boronic acids as reported in the literature.[17]

B,B',B''-tri(4-bromophenyl)borazine

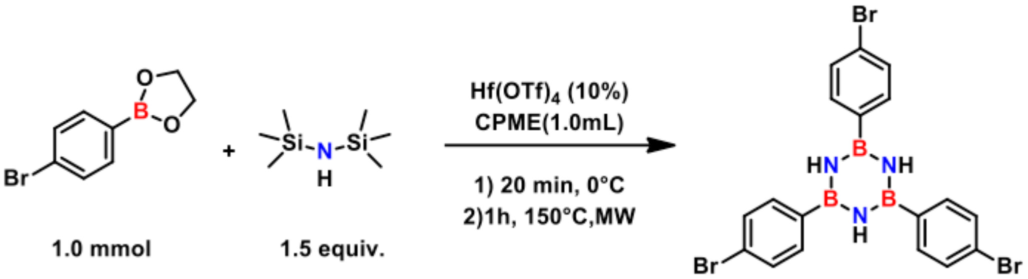

An oven-dried microwave vessel (10 mL) was charged with freshly synthesized 2-(4-bromophenyl)-1,3,2-dioxaborolane (1.0 mmol, 227 mg) and dried $Hf(OTf)_4$ (10%, 77 mg). After an evacuate-refill cycle, dried cyclopentyl methyl ether (CPME, 1.0 mL, freshly distilled from CaH_2) and hexamethyldisilazane (HMDS, 1.55 mmol, 325 µL) were added at 0°C, and the mixture was stirred at the same temperature for 20 minutes. The reaction vessel was then placed in a microwave synthesizer and heated at 150°C for 1 hour under 300 psi and 150 W. After cooling down, the reaction mixture was dissolved in diethyl ether (10 mL), and $Hf(OTf)_4$ was removed by filtration. The filtrate was evaporated, and the crude product was washed with hexane to yield an off-white solid as the pure product (135 mg, 74%). Melting point: 275ºC, ^{1}H NMR (400 MHz , $CDCl_3$) δ 7.64-7.58(m, 12H) and 5.80 (s, 3H, N-H). ^{13}C NMR (100.6 MHz, $CDCl_3$) δ: 133.66, 131.59, 125.07 (One peak is missing due to quadrupolar B-induced relaxation).

***B,B',B''*-tri(4'-bromo-[1,1'-biphenyl]-4-yl)borazine**

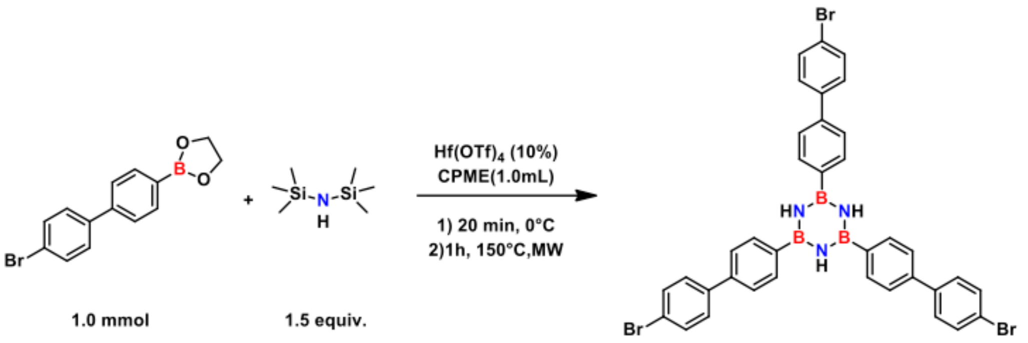

An oven-dried microwave vessel (10 mL) was charged with freshly synthesized 2-(4'-bromo-[1,1'-biphenyl]-4-yl)-1,3,2-dioxaborolane (1.0 mmol, 303 mg) and dried $Hf(OTf)_4$ (10%, 77 mg). After an evacuate-refill cycle, dried cyclopentyl methyl ether (CPME, 1.0 mL, freshly distilled from CaH_2) and hexamethyldisilazane (HMDS, 1.55 mmol, 325 µL) were added at 0°C, and the mixture was stirred at the same temperature for 20 minutes. The reaction vessel was then placed in a microwave synthesizer and heated at 150°C for 1 hour under 300 psi and 150 W. After cooling down, the reaction mixture was dissolved in diethyl ether (10 mL), and $Hf(OTf)_4$ was removed by filtration. The filtrate was evaporated, and the crude product was extensively washed with hexane to yield an off-white solid as the pure product (90 mg, 35%). Melting point: > 300ºC, ^{1}H NMR (400 MHz, $CDCl_3$) δ 7.89 (d, *J*=7.7Hz, 6H), 7.68 (d, *J*=7.8Hz, 6H), 7.60 (d, *J*=8.3Hz, 6H), 7.53 (d, *J*=8.3Hz, 6H), and 5.98 (s, 3H, N-H). ^{13}C NMR (100.6 MHz, $CDCl_3$) δ: 141.74, 139.95, 132.77, 132.13, 128.91, 126.89, 122.04 (One peak is missing due to quadrupolar B-induced relaxation).

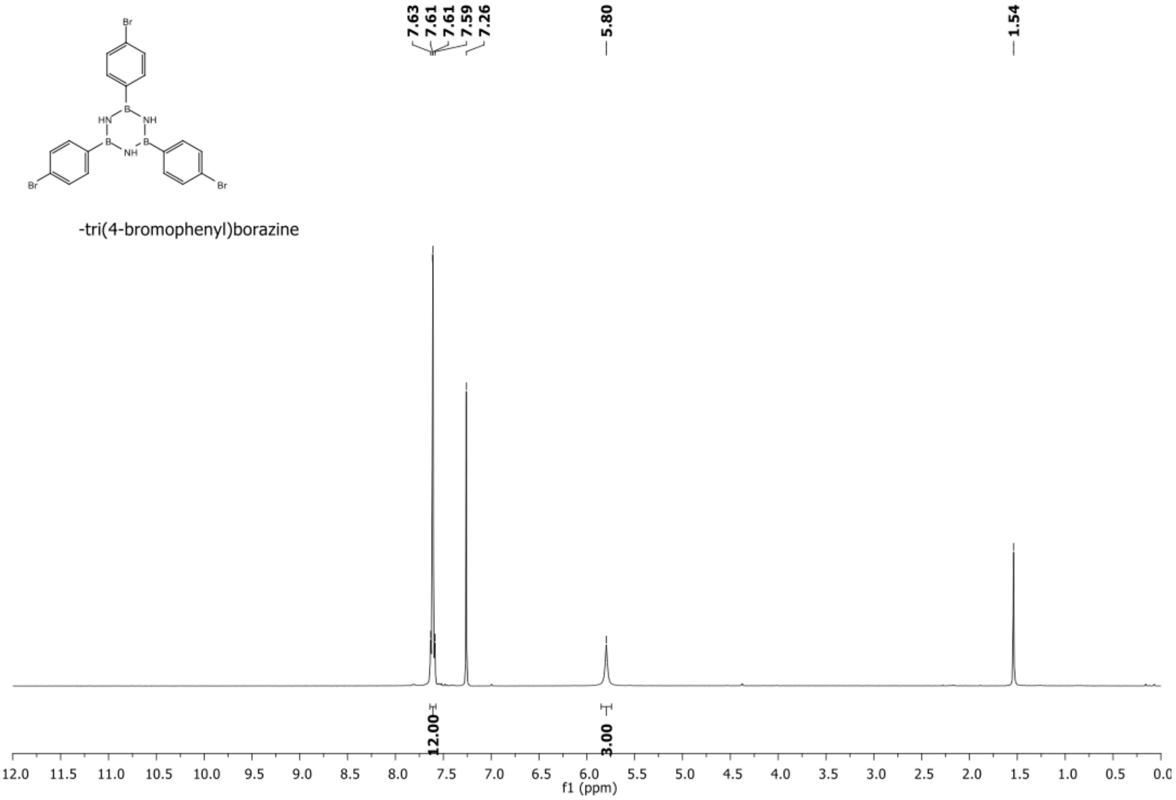

-tri(4-bromophenyl)borazine

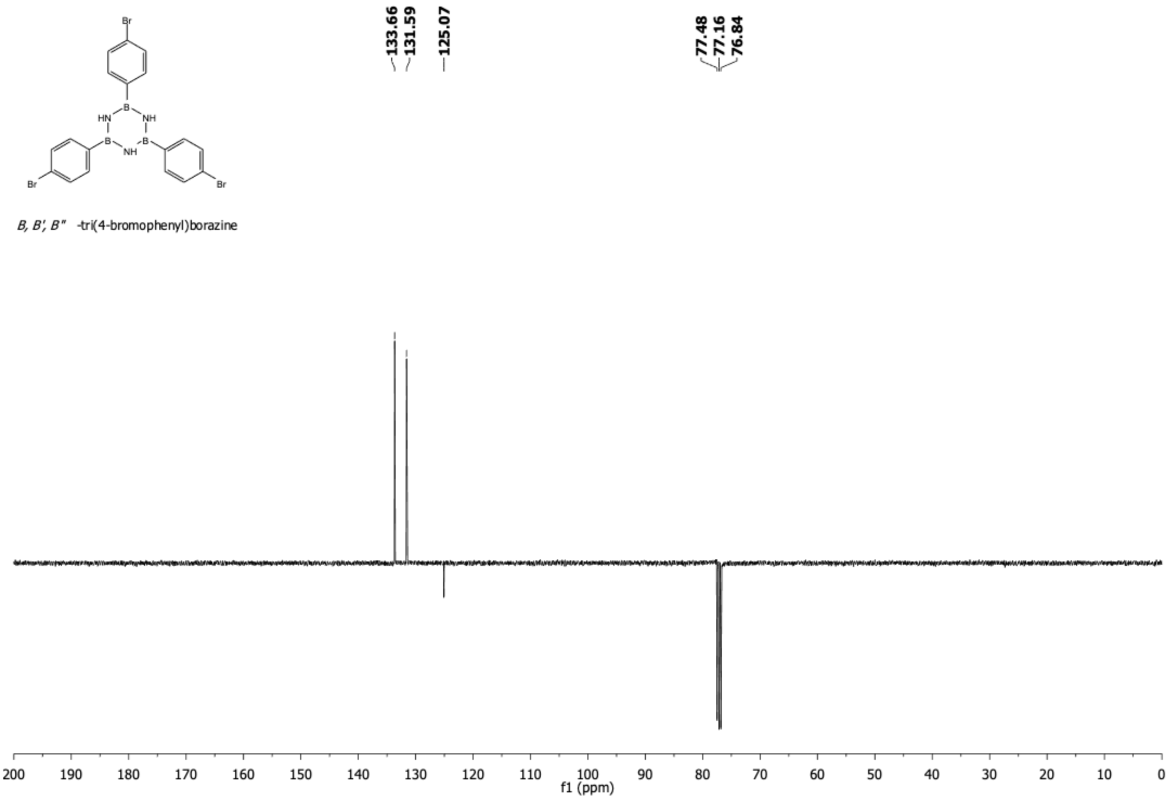

B, B', B'' -tri(4-bromophenyl)borazine

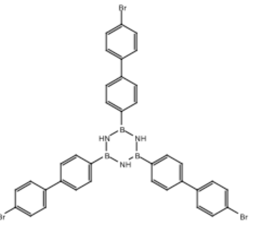

''-tri(4'-bromo-[1,1'-biphenyl]-4-yl)borazine

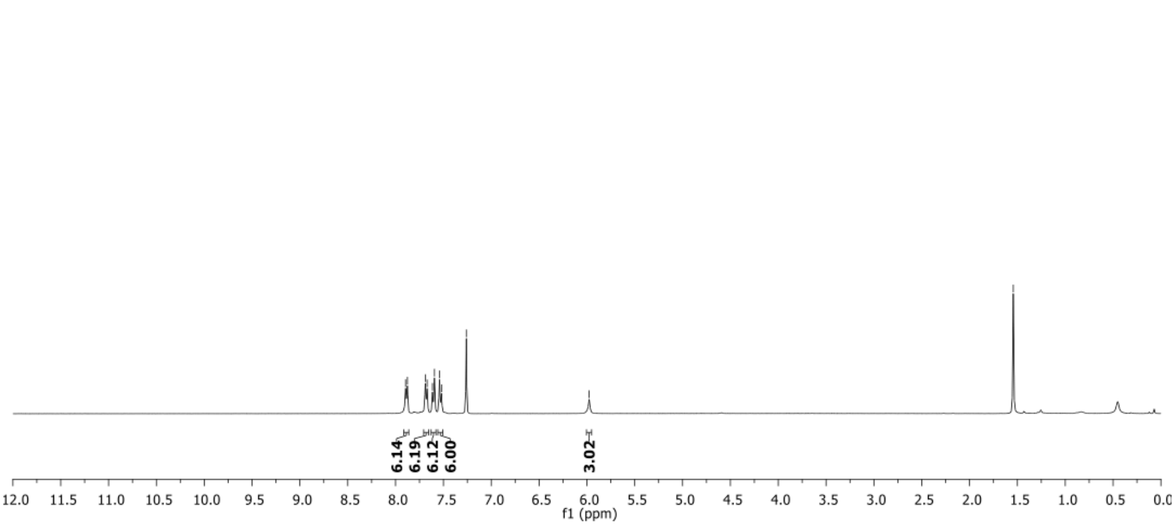

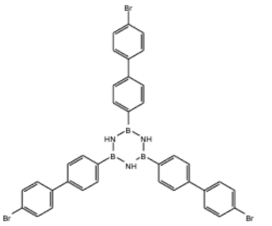

B, B', B''-tri(4'-bromo-[1,1'-biphenyl]-4-yl)borazine

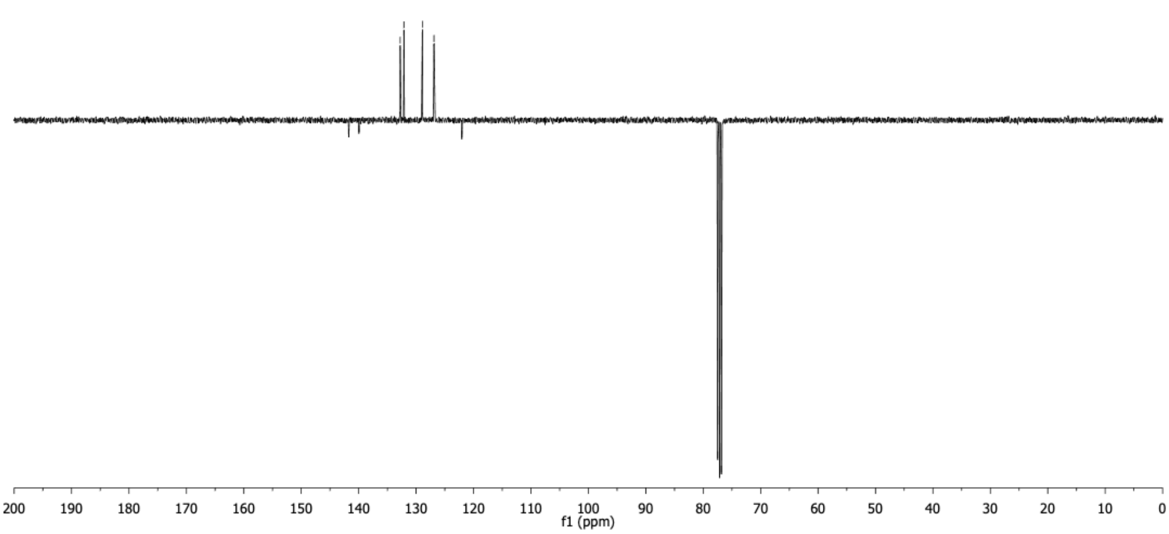

REFERENCES

[1] I. Horcas, R. Fernández, J. M. Gómez-Rodríguez, J. Colchero, J. Gómez-Herrero, A. M. Baro, *Rev. Sci. Instrum.* **2007**, *78*, 013705.

[2] M. J. van Setten, M. Giantomassi, E. Bousquet, M. J. Verstraete, D. R. Hamann, X. Gonze, G. M. Rignanese, *Comput. Phys. Commun.* **2018**, *226*, 39–54.

[3] P. Giannozzi, O. Andreussi, T. Brumme, O. Bunau, M. B. Nardelli, M. Calandra, R. Car, C. Cavazzoni, D. Ceresoli, M. Cococcioni, *et al.*, *J. Phys. Condens. Matter* **2017**, *29*, 465901.

[4] J. P. Perdew, K. Burke, M. Ernzerhof, *Phys. Rev. Lett.* **1996**, *77*, 3865–3868.

[5] A. Otero-de-la-Roza, E. R. Johnson, V. Luaña, *Comput. Phys. Commun.* **2014**, *185*, 1007–1018.

[6] A. Otero-de-la-Roza, M. A. Blanco, A. M. Pendás, V. Luaña, *Comput. Phys. Commun.* **2009**, *180*, 157–166.

[7] F. Jollet, M. Torrent, N. Holzwarth, *Comput. Phys. Commun.* **2014**, *185*, 1246–1254.

[8] P. Hapala, R. Temirov, F. S. Tautz, P. Jelínek, *Phys. Rev. Lett.* **2014**, *113*, 1–5.

[9] P. Hapala, G. Kichin, C. Wagner, F. S. Tautz, R. Temirov, P. Jelínek, *Phys. Rev. B - Condens. Matter Mater. Phys.* **2014**, *90*, 085421.

[10] H. Hövel, B. Grimm, B. Reihl, *Surf. Sci.* **2001**, *477*, 43–49.

[11] I. Piquero-Zulaica, J. Lobo-Checa, Z. M. A. El-Fattah, J. E. Ortega, F. Klappenberger, W. Auwärter, J. V. Barth, *Rev. Mod. Phys.* **2022**, *94*, 045008.

[12] X, R. Gutzler, *Phys. Chem. Chem. Phys.* **2016**, *18*, 29029–29100.

[13] J. Van Der Lit, M. P. Boneschanscher, D. Vanmaekelbergh, M. Ijäs, A. Uppstu, M. Ervasti, A. Harju, P. Liljeroth, I. Swart, *Nat. Commun.* **2013**, *4*, 2023.

[14] Z. Majzik, A. B. Cuenca, N. Pavliček, N. Miralles, G. Meyer, L. Gross, E. Fernández, *ACS Nano* **2016**, *10*, 5340–5345.

[15] F. Bischoff, A. Riss, G. S. Michelitsch, J. Ducke, J. V. Barth, K. Reuter, W. Auwärter, *J. Am. Chem. Soc.* **2021**, *143*, 15131–15138.

[16] N. Bassi, X. Xu, F. Xiang, N. Krane, C. A. Pignedoli, A. Narita, R. Fasel, P. Ruffieux, *Commun. Chem.* **2024**, *7*, 274.

[17] G. Ranjani, R. Nagarajan, *Org. Lett.* **2017**, *19*, 3974–3977.
